# Tau-mediated coupling between Pol III synthesis and DnaB helicase unwinding helps maintain genomic stability

**DOI:** 10.1016/j.jbc.2024.107726

**Published:** 2024-08-29

**Authors:** Malisha U. Welikala, Lauren J. Butterworth, Megan S. Behrmann, Michael A. Trakselis

**Affiliations:** Department of Chemistry and Biochemistry, Baylor University, Waco, Texas, USA

**Keywords:** coupling, decoupling, Tau, clamp loader, DNA helicase, unwinding, DNA replication, genomic instability

## Abstract

The τ-subunit of the clamp loader complex physically interacts with both the DnaB helicase and the polymerase III (Pol III) core α-subunit through domains IV and V, respectively. This interaction is proposed to help maintain rapid and efficient DNA synthesis rates with high genomic fidelity and plasticity, facilitating enzymatic coupling within the replisome. To test this hypothesis, CRISPR-Cas9 editing was used to create site-directed genomic mutations within the *dnaX* gene at the C terminus of τ predicted to interact with the α-subunit of Pol III. Perturbation of the α-τ binding interaction *in vivo* resulted in cellular and genomic stress markers that included reduced growth rates, fitness, and viabilities. Specifically, *dnaX:mut* strains showed increased cell filamentation, mutagenesis frequencies, and activated SOS. *In situ* fluorescence flow cytometry and microscopy quantified large increases in the amount of ssDNA gaps present. Removal of the C terminus of τ (I618X) still maintained its interactions with DnaB and stimulated unwinding but lost its interaction with Pol III, resulting in significantly reduced rolling circle DNA synthesis. Intriguingly, *dnaX:L635P/D636G* had the largest induction of SOS, high mutagenesis, and the most prominent ssDNA gaps, which can be explained by an impaired ability to regulate the unwinding speed of DnaB resulting in a faster rate of *in vitro* rolling circle DNA replication, inducing replisome decoupling. Therefore, τ-stimulated DnaB unwinding and physical coupling with Pol III acts to enforce replisome plasticity to maintain an efficient rate of synthesis and prevent genomic instability.

DNA replication in *Escherichia* *coli* has been well studied and shown to consist of a minimal replisome composed of at least 13 proteins that function together to ensure faithful and accurate replication of DNA (1). The main replicative helicase, DnaB, translocates in the 5’ – 3′ direction and unwinds double stranded DNA by encircling the lagging strand and sterically excluding the leading strand (2). The polymerase III (Pol III) holoenzyme consists of the core (α, ε, and θ subunits), which synthesize nascent DNA leading and lagging strands, a β clamp that interacts with Pol III to increase the processivity, and the clamp-loader complex (CLC) to load the β clamp (3). The CLC plays an important role as the central organizer of the replisome, coordinating clamp loading, coupling unwinding and synthesis, and maintaining equal rates for leading and lagging strand synthesis (4, 5).

Recent advances in single molecule technology both *in vitro* and *in vivo* have shown that the bacterial replisome is surprisingly dynamic with frequent exchanges of polymerases and accessory subunits in a concentration-dependent manner, while the helicase remains a stable hub for the assembly of the rest of the replisome proteins (6, 7, 8). There is a dichotomy within the replisome between a more stable helicase complex as an anchor for unwinding and the more stochastic behavior of Pol III required for the discontinuous synthesis of Okazaki fragments or traversing challenging regions for synthesis (9). Therefore, a balance in maintaining continuity or coupling of the DNA unwinding and synthesis processes despite their differential dynamic equilibria processes must be maintained, highlighting significant variability and heterogeneity in replisomes (10). In the *E. coli* replisome, this continuity is thought to be maintained through a physical link between the polymerase and helicase provided by the τ-subunit of the CLC that helps regulate replication speed (Fig. 1*A*) (11).

**Figure 1:**
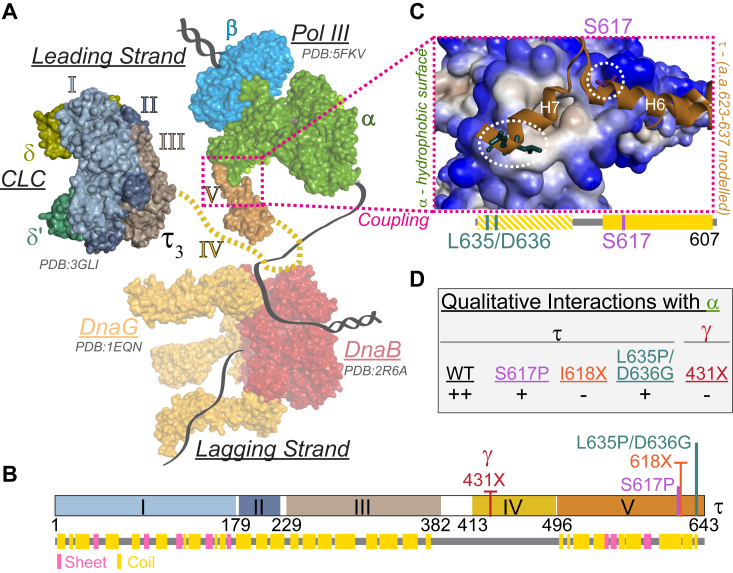
**Schematic of the structure of τ-subunit of the clamp loader complex interacting within the bacterial replisome.***A*, X-ray crystal structures of DnaB (PDBID: 2R6A, *red*), DnaG (PDBID: 1EQN, *orange*), Pol III-β-τ (PDBID: 5FKV, α, ε, θ, *green*; β, *cyan*; τ; *orange*,), and γ-CLC (PDBID: 3GLI, Domains I, *light steel blue*, II, *Bermuda grey*, III, *akaroa*) were used to model a proposed leading strand bacterial replisome that physically couples unwinding and synthesis. Unstructured domain IV of τ is shown as dashed line (*mustard*). *B*, linear domain structure (I-V) of τ, highlighting the γ-truncation (431X, *red*), mutations (S617P, *pink*; L635P/D636G, *teal*), and the truncation at 618X (*orange*). The secondary structure sheet (*pink*) and coil (*yellow*) are indicated below based on the available structure and PSIPRED algorithm. *C*, a closeup of the proposed coupling interaction of domain V of τ interacting with a hydrophobic surface representation of α is highlighted with a modeled extension of residues 523 to 536 based on the predicted secondary structure (*yellow* hashed). *D*, qualitative assessment of the extent of interaction between His tagged-α subunit of Pol III and respective τ_3_-CLC proteins from a pull-down assay (Fig. S1).

The *dnaX* gene encodes for both τ and γ subunits; however, γ is a truncated form of τ at residue 431 early within domain IV, as a result of programmed translational frameshifting (Fig. 1*B*) (12, 13). Domains IV and V are present in τ but absent in γ and provide for the connection between DnaB and the α subunit of Pol III, respectively. Residues 430 to 498 within domain IV are responsible for binding DnaB (11), while domain V and specifically C-terminal residues, 611 to at least 636, interact with α (14, 15, 16). Structured globular domains I-III within the core of the CLC are connected to domains IV/V through an unstructured long linker, giving flexibility and mobility for τ to interact with other proteins and respond to dynamic events occurring within the replisome. The CLC can contain two to three τ subunits (along with δδ′ψχ) (15, 16). Therefore, the leading and lagging strand Pol IIIs can be linked through two τ subunits within the same CLC (17), and DnaB can be linked to the leading strand Pol III through one of those τ subunits (5). A third τ subunit can be utilized to recruit a third Pol III core in waiting for loading onto the next RNA primer for further Okazaki fragment synthesis *in vitro* (11), although genetic and cellular evidence favors a native τ_2_γ_1_-CLC *in vivo* (18, 19, 20, 21).

The τ-mediated interaction between DnaB and Pol III enables the rate of coupled DNA unwinding and synthesis to approach ∼1000 nt/s (5). In the absence of this coupling interaction, a molecular switch in the conformation of DnaB causes the translocation/unwinding rate to be reduced to ∼35 nt/s (22). Uncoupling between the DnaB and Pol III can result in continued unwinding by the helicase but at a reduced rate of synthesis, leading to the accumulation of intervening ssDNA gaps (23). Blocks to replication may be caused by DNA damage, interfering transcription machinery, or tension in the supercoiled template (24, 25). Specifically, blocks to leading strand synthesis may result in decoupling with DnaB as unwinding/translocation continues on the lagging strand. Should replisome decoupling occur, an excess of ssDNA can be filamented by RecA triggering an SOS response that induces the expression of error prone DNA polymerases and other DNA repair enzymes attempting to mitigate this stress (23, 26).

For DNA replication to occur with high fidelity and efficiency, it is imperative that the synthesis of nascent strands occur immediately after DNA unwinding, thus reducing accumulation of labile ssDNA that could cause significant genomic instability. To further understand the genomic and cellular importance of this replisome coupling phenomenon, *dnaX* mutations corresponding to specific amino acid changes in the C-terminal helix-turn-helix motif of τ and predicted to weaken the induced interaction with the α-subunit of Pol III (15) were created (Figs. 1, *B* and *C* and S1). *dnaX*:*mut* strains showed significant increases in cellular stress that included extreme cellular elongation, reduced growth rates, compromised fitness, increased SOS, and increased mutagenesis. In particular, *dnaX*:*L635P/D636G* displayed more deleterious effects overall than *dnaX*:*S617P*. Although all τ_3_-CLC mutants stimulated DnaB unwinding in isolation and τ_3_-CLC(S617P) and τ_3_-CLC(L635P/D636G) performed leading strand synthesis at rates similar (or even greater) to WT, their ability to dilate DnaB to traverse over duplex DNA was impaired. This regulation switch of τ_3_-CLC in coordinating both Pol III recruitment and regulating DnaB unwinding plays an important role in maintaining efficient replisomal coupling of DNA unwinding and synthesis. Altogether, at least the C-terminal 26 residues of τ coordinate unwinding and synthesis within the replisome to maintain genomic stability in the bacterial cell.

## Results

### dnaX mutations limit growth, fitness, and viability

Previous work mapping the τ-α interaction concluded that upon binding, contacts occur through a structured helix (H6) which may induce previously unstructured residues 624 to 635 to adopt an additional downstream α-helix (H7), creating a helix-turn-helix motif (Fig. 1*C*) (15). They further concluded that these last 18 residues of τ (a.a. 626–643) are utilized for the interaction with α, in which the last 11 (a.a. 626–636) are required and the last 7 (a.a. 630–636) are influential. Within that stretch of amino acids, they also identified several suppressor mutations to the growth inhibition found by overexpressing domains IV-V that allowed binding to α with modest changes to binding free energy (ΔΔG), including S617P (4.1 kcal/mol) and L635P or D636G (1.7 or 1.9 kcal/mol each). Before creating CRISPR/Cas9 edits in the *dnaX* gene to test replisome coupling, we made and tested full-length mutants of τ: S617P, L635P/D636G, and truncated τ, I618X, for qualitative binding interactions with the α-subunit of Pol III using nickel pull-down assays. The intensity of the protein bands coeluting were quantified to obtain fractional saturation values as a ratio of τ (or γ) to α bands (Fig. 1*D* & S1). WT τ showed the highest fractional saturation value, indicating the strongest interaction with α, while L635P/D636G and S617P had reduced fractional saturation values, exhibiting weaker interactions with α. The control C-terminal truncation, I618X, deleting the last 25 amino acids, showed negligible binding with α, similar to γ, consistent with previous more quantitative analyses (15).

These same τ protein mutations were genomically edited in *dnaX* using CRISPR/Cas9 in the parental strain, MG1655. To increase the efficiency of recombination of a synthetic oligonucleotide containing the desired mutation and a novel restriction site for screening, the λ-red genes were exogenously induced. An outright deletion of the C-terminal residues of τ (*dnaX:I618X*) seemed likely to fail based on this previous evidence for its importance (15) and was evidenced by our several unsuccessful attempts to isolate this strain. However, several successfully edited mutated colonies were produced for (*dnaX*:*S617P* and *dnaX:L635P/D636G*) as screened by colony PCR and restriction digest (Fig. S2). These *dnaX*:*mut* strains were cured of pRedCas9 by growth at 42 °C to target the temperature-sensitive origin, p15A to isolate the new strains. The strains were confirmed by whole genome sequencing to contain only the desired mutations and not any other obvious suppressors (Table S4).

**Figure 2:**
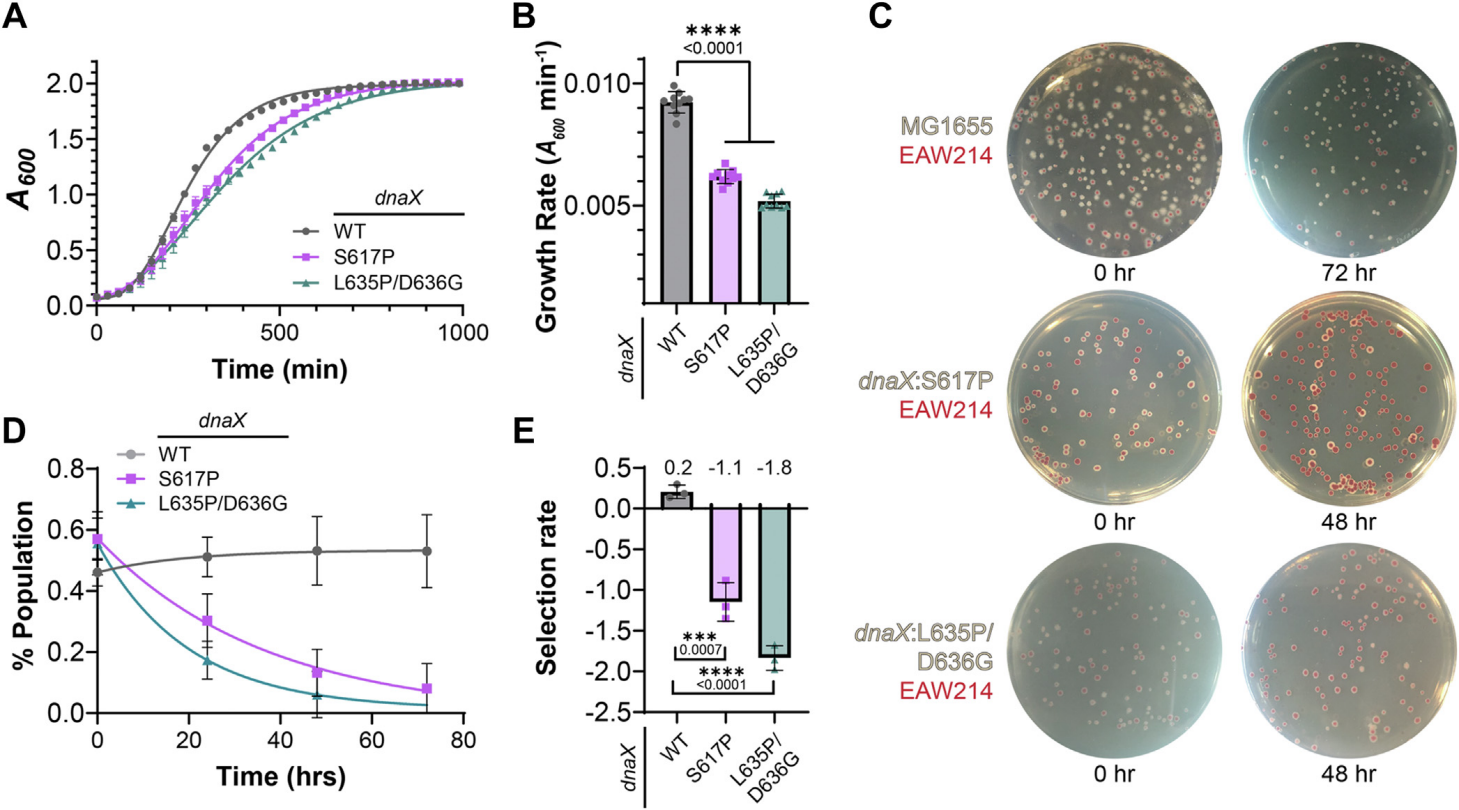
***dnaX*:*mut* strains show reduced growth rates and fitness.***A*, growth curve of *dnaX* strains (*WT* - • *gray*, *S617P* - ▪ *lavender*, *L635P/D636G* - ▲ *teal*) at 37 °C monitored using absorbance at A_600_. Data plotted is the averaged growth profiles for 10 technical replicates. *B*, the absolute growth rates (*k*_*z*_) were calculated from Equations (1), (2), and the average was plotted as a bar. *C*, a colorimetric competition assay in which EAW214 (*ΔaraBAD*) and each of the *dnaX* strains were mixed and allowed to compete for growth. The arabinose present in the plates reduces the dye triphenyl tetrazolium chloride, giving a *red* color for EAW214 (Ara^-^), while the other *dnaX* strain colonies appear *white* in color (Ara^+^) with representative plates shown for each. *D*, the changes in the population of mixed cultures were plotted as a function of time for a minimum of three biological replicates with error bars indicating SE and fit to an exponential decay. *E*, the selection rates for each of the cultures were calculated using Equation 3 and plotted. *Black* bars indicate statistically significant differences with *p*-values indicated and represented by ∗∗∗<0.001 and ∗∗∗∗<0.0001 from an unpaired two-sided *t* test.

Mass doubling times for all *dnaX*:*mut* strains were monitored at *A*_*600*_ in 96-well plates using a plate reader at 37 °C. The growth rates can be suboptimal using this method which restricts aeration, but the assay is internally controlled and can be conveniently monitored and compared. The absolute growth rate (*k*_*z*_) for the parental MG1655 stain is 9.2 ± 0.4 × 10^−3^ min^−1^ and is consistent with previous studies using other instruments and strains (27). However, the rates for both *dnaX*:*S617P* (6.2 ± 0.3 × 10^−3^ min^−1^) and *dnaX*:*L635P/D636G* (5.2 ± 0.3 × 10^−3^ min^−1^) are significantly reduced (Fig. 2, *A* and *B*).

As the *dnaX*:*muts* had slower overall growth rates, we hypothesized that their relative fitness compared to the parental strain would also be reduced. To compare the fitness rates between two strains, a colorimetric growth competition assay was performed. A parental strain with a deletion of the arabinose isomerase promoter (EAW214: MG1655:ΔaraBAD) turns red from the inability to utilize the reducing sugar, arabinose, which converts triphenyltetrazolium chloride to red formazan on the agar plates (27, 28). On the same plates, *dnaX*:*muts* (*ara*^*+*^) degrade arabinose, leaving triphenyltetrazolium chloride oxidized (colorless) and produce typical white colonies (Fig. 2*C*). As an initial control, EAW214 (*ara*^-^) and MG1655 (*ara*^+^) strains were cocultured and plated on successive days and show consistently equal populations over 48 to 72 h, while both *dnaX*:*mut* strains declined in population relative to EAW214 (Fig. 2*D*). In fact, to maintain an equal population of colonies on day 0, a 4:1 mixture ratio of *dnaX*:*L635P/D636G* to EAW214 was required for the initial coculture. The selection rates for each of the strains were quantified where *dnaX*:*S617P* and *dnaX*:*L635P/D63G* had negative selection rates of −1.1 ± 0.1 and −1.8 ± 0.1, respectively, indicating severely reduced fitness compared to parental strains (Fig. 2*E*).

### dnaX:muts show signs of cellular and genomic stress

**Figure 3:**
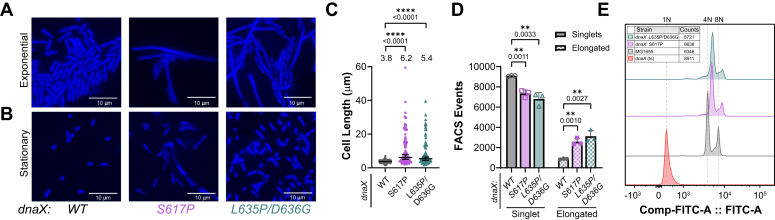
**Cell filamentation and chromosome complexity is increased in *dnaX*:*mut* strains.** (*A*) Exponential or (*B*) stationary phase cells stained with DAPI are imaged using an epifluorescence microscope. *C*, cell lengths from exponential populations were measured (n > 80) and median values were plotted. The error bars denote 95% confidence limits, and *black* bars indicate the statistically significant differences from *p*-values of ∗∗∗∗ <0.0001. *D*, the singlet and elongated gate populations from FACS are plotted from three biological replicates. *Black* bars indicate statistically significant differences with *p*-values indicated and represented by ∗∗<0.01 from an unpaired two-sided *t* test. *E*, chromosome complexity observed in *dnaX*:*mut* strains treated with rifampicin and cephalexin were runout, stained with Sytox Green, and then analyzed using FACS (n = 10,000 events). The fluorescence intensity *versus* counts was plotted as histograms. A temperature sensitive *dnaA*:(ts) strain was used to define the location of cells with single chromosomes along with four or eight chromosomes in the parental cells and are indicated at the *top* of the graph.

As a phenotypic consequence, cellular or genomic stress can be reflected as changes in cell size or lengths. To confirm and quantify whether *dnaX*:*mut* cells have altered cellular morphologies, exponential and stationary phases were stained with 4′,6-diamidino-2-phenylindole (DAPI) and imaged. In exponential growing cells, both mutants, *dnaX*:*S617P* and *dnaX*:*L635P/D63G,* showed significantly elongated cells with median cell sizes of 6.2 and 5.4 μm respectively compared to the parental strain with a median cell size of 3.8 μm (Fig. 3, *A* and *C*). Interestingly, there were several cells with vastly elongated lengths of greater than 30 μm in each of the *dnaX*:*mut* strains. In stationary phase cells, the median cell length for the parental strain reduced to 1.8 μm, while the median cell lengths of *dnaX*:*S617P* and *dnaX*:*L635P/D63G* in stationary phase were only reduced to 4.7 and 3.0 μm respectively and were significantly longer than the parental strain (Fig. 3*B* & S3). To more globally assess cellular filamentation, we utilized FACS to quantify the subpopulations of singlets or elongated cells out of 10,000 events (Fig. 3*D*). Interestingly, both *dnaX:muts* show significant increases in the proportion of elongated cells, 26.0% for *dnaX*:*S617P* and 31.4% for *dnaX*:*L635P/D63G* compared to the parental strain of 8.8%, similar to the microscopy results.

Cell filamentation observed in *dnaX*:*mut* strains may indicate inefficient replisome activity or genomic stress and result in a delay in fission. Generally, synchronized bidirectional replication in *E. coli* will result in *2n* number (*n* being any integer value) of chromosomes in exponentially growing cells (29). Treatment with rifampicin and cephalexin inhibits initiation of replication and cell division, respectively. A growth runout for 4 h gives cells sufficient time to complete ongoing rounds of replication before analysis of chromosome content by FACS (Figs. 3*E* and S4) (30). In the MG1655 strain, two major peaks are observed, correlating to four and eight number of chromosomes; however, *dnaX*:*mut* strains contain a minor peak less than four chromosomes, indicating a delay in synthesis; a major peak greater than four chromosomes, indicating asynchronous replication initiation; and a minor broader peak corresponding to 8+ chromosomes, indicating overreplication or severe chromosomal excess or entanglement. Therefore, both *dnaX*:*S617P* and *dnaX*:*L635P/D63G* induce significant challenges for efficient and synchronous DNA replication that result in grossly abnormal chromosomal content.

### Induction of SOS causes increased mutagenesis in dnaX:mut strains

**Figure 4:**
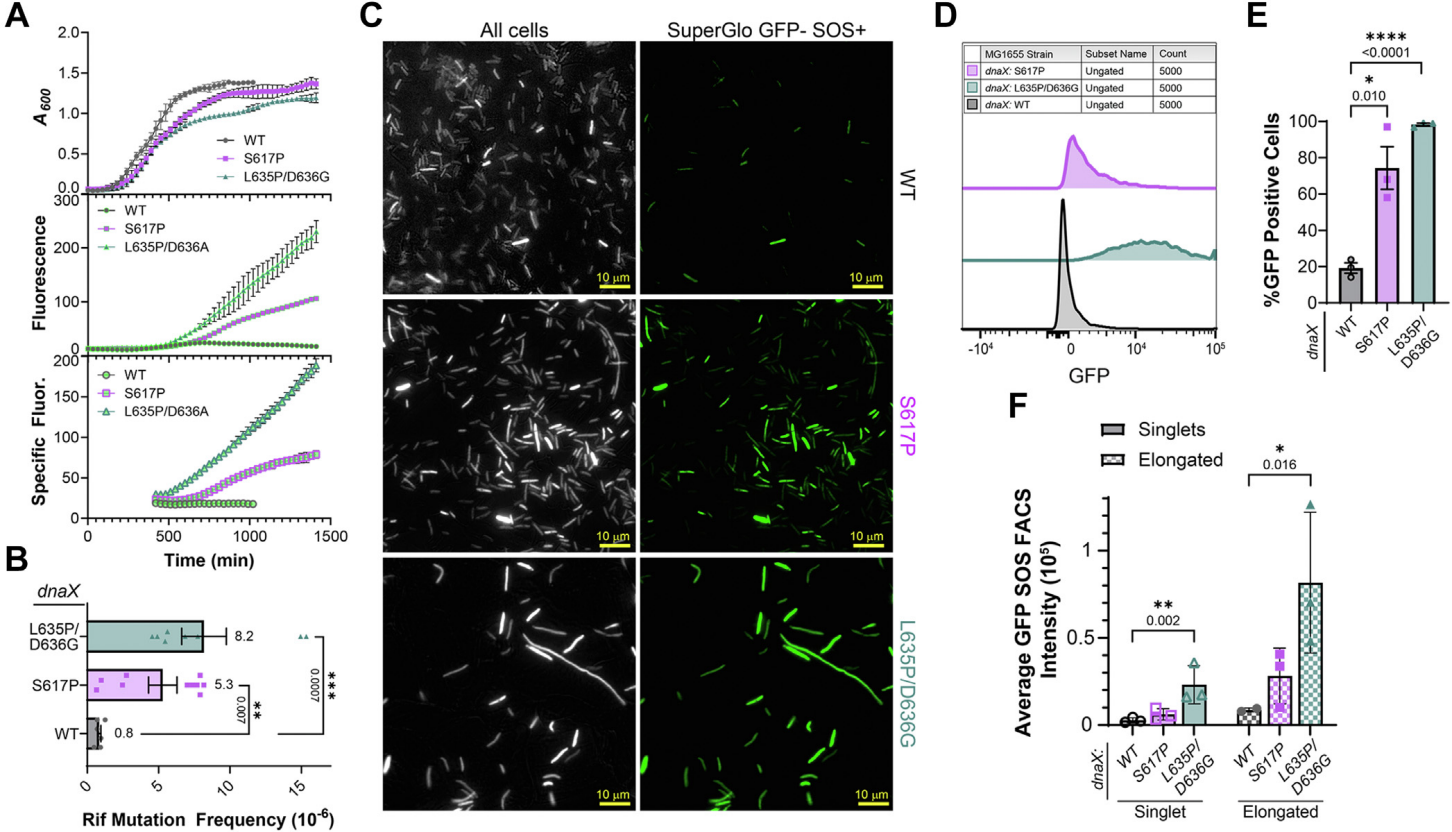
***dnaX*:*mut* strains induce SOS and increased mutagenesis.***A*, the growth (A_600_) and fluorescence (ex 474 nm/em 509 nm) from induction of sgGFP was monitored for *dnaX*:*mut* strains in Miller LB media using a plate reader allowing the calculation of specific fluorescence (*bottom* panel) using Equation 4. *B*, strains were plated on ± rifampicin (rif) Miller LB plates and cfu were quantified. Mutagenesis frequency for each of the strains was calculated using Equation 5. The average number of mutants per 10^6^ cfu were plotted with the SE indicated. *Black* bars indicate statistically significant differences where *p*-values are indicated and represented by ∗∗<0.01 or ∗∗∗<0.001, from a Mann-Whitney *U*-test. Stationary phase populations of strains expressing sgGFP were examined by (*C*) microscopy or (*D*) FACS (n = 5000 events, each for three biological replicates) and (*E*) quantified for ungated %GFP positive cells. *F*, the average GFP fluorescence intensity per cell measured by FACS was also plotted for gated singlets or elongated cells with the SE indicated. *Black* bars indicate statistically significant differences, where *p*-values are indicated and represented by ∗<0.05, ∗∗<0.01, or ∗∗∗∗<0.0001, from an unpaired two-sided *t* test.

Disruption in replisome coupling can lead to a large accumulation of ssDNA that triggers an induction of the SOS response to promote cellular survival (25). This SOS response results in the expression of a set of genes that work to delay cell division to repair any DNA damage (31). To determine whether the *dnaX*:*mut* strains induce SOS created from uncoordinated helicase unwinding and polymerase synthesis even in the absence of DNA damaging agents, they were transformed with a vector expressing SuperGlo GFP (sgGFP) under control of the *recN* promoter to monitor SOS induction (32). Strains were grown in the absence (Fig. 4*A*) or presence (Fig. S5) of a low dose of mitomycin C (MMC), and the specific fluorescence was calculated (30). Specific fluorescence is used to account for the increase in cell density due to growth over time. The *dnaX*:*mut* strains exhibited dramatic increases in SOS induction in both the absence and presence of MMC compared to the parental strain. *dnaX*:*L635P/D636G* showed the highest specific fluorescence over time in both nontreated and MMC-treated conditions, but *dnaX*:S617P also showed a substantial increase in GFP signal associated with SOS in both scenarios. *dnaX*:*WT* as expected had minimal specific fluorescence over time in nontreated conditions and increased slightly over time under the low dose MMC treatment.

As SOS induction also facilitates an increase in mutagenicity by increased expression of error prone DNA polymerases, we wanted to examine the mutational frequencies of these *dnaX*:*mut* strains after exposure to rifampicin. Mutations arising in the *rpoB* gene, encoding for the β subunit of RNA polymerase, confers resistance to rifampicin (33). By quantifying the frequency of rifampicin mutagenesis, the mutagenicity of strains during heightened SOS can be determined (34). The parental strain had an average of 0.8 ± 0.1 mutation events per 10^6^ number of cells (Fig. 4*B*), consistent with previous results (27). However, both mutant strains, *dnaX*:*S617P* and *dnaX*:*L635P/D636G*, showed significantly higher mutation rates than the parental strain with 8.2 ± 1.6 and 5.3 ± 1.0 mutation events per 10^6^ cells, respectively.

To better visualize the abundance of SOS in untreated exponential cell populations, we examined sgGFP expression by microscopy and FACS. Both *dnaX*:*S617P* and *dnaX*:*L635P/D636G* showed significant GFP expression in most all cells, independent of an obvious subpopulation that were filamented (Fig. 4*C*). FACS was used to quantify GFP expression in cells and showed that both *dnaX*:*S617P* (74 ± 12%) and *dnaX*:*L635P/D636G* (98 ± 1%) had a significantly higher number of cells that were GFP-positive compared to *dnaX:WT* (19 ± 3%) (Fig. 4, *D* and *E*). Interestingly, not only were the fraction of GFP-positive cells increased in both the singlets and elongated cells, but the average absolute fluorescence intensities were also increased in those subpopulations, indicating a massive increase in SOS induction (Figs. 4*F* and S6).

### Increased ssDNA gaps are present in dnaX:mut strains

**Figure 5:**
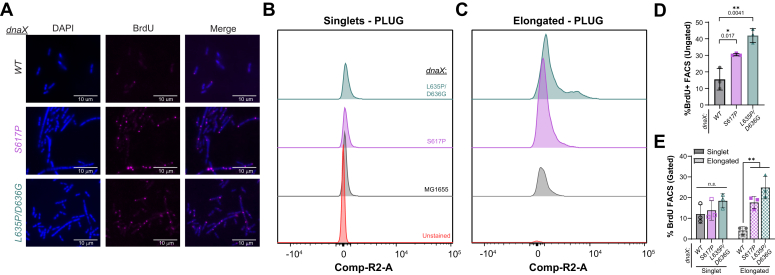
**Increased ssDNA gaps are present in elongated *dnaX*:*mut* cells.***A*, exponential phase cells were treated with Klenow polymerase and BrdU *in situ*, probed for BrdU in a PLUG assay, and imaged using immunofluorescence microscopy. *Blue* – DAPI panel represents DNA staining and *pink* – BrdU represents ssDNA gaps. The fluorescence intensity of the cells for BrdU positive were also quantified using FACS (n = 10,000 events, each for three biological replicates) and the histograms for gated cells are shown (*B*) for singlets and (*C*) elongated cells. Quantification of the %BrdU positive PLUG cells for the (*D*) ungated population or (*E*) comparing singlets and elongated cells are plotted with the SE indicated. *Black* bars indicate statistically significant differences, where *p*-values are indicated and represented by ∗<0.05 or ∗∗<0.01 from an unpaired two-sided *t* test.

As the SOS response is a consequence of accumulation of significant ssDNA gaps coated by RecA, a Pol I dUTP Gap filling (PLUG) assay was utilized to visualize the ssDNA gaps *in situ* (23, 35). Fixed and permeabilized cells were treated with Klenow fragment (Polymerase I – 5’ - 3′ exonuclease deficient variant) and nucleotides that included 5-bromo-2′-deoxyuridine (BrdU) that allowed gap filling extension to occur. Afterwards, filled gaps were marked by immunofluorescent probing of BrdU incorporation. For qualitative measurements, cells were visualized by fluorescent microscopy and both *dnaX*:*mut* strains appeared to show a greater number of BrdU foci, representing ssDNA gaps, when compared to *dnaX*:*WT* (Fig. 5*A*). To more thoroughly quantify BrdU labeling, the cells were analyzed by flow cytometry. Altogether, the ungated cell populations of *dnaX*:*S617P* and *dnaX*:*L635P/D636G* had 30.7 and 42.3% of the cells labeled for BrdU, respectively, compared to only 15.6% of the WT population (Fig. 5*D*). Because both *dnaX*:*S617P* and *dnaX*:*L635P/D636G* showed increased cell filamentation (Fig. 3, *A*–*D*), BrdU-positive cells were gated and categorized into two populations: singlets and elongated (Figs. 5, *B*–*E* & S7). Of these singlet events, 12.0% of *dnaX*:*WT* cells were positive for BrdU, and both *dnaX*:*S617P* and *dnaX*:*L635P/D636G* had slightly increased BrdU positive percentages for singlet cells of 13.8% and 18.4% respectively (Fig. 5*E*). Interestingly, *dnaX*:*S617P* and *dnaX*:*L635P/D636G* had significantly greater percentages of BrdU-positive elongated cells (17.6 and 24.9% of the total cell population) compared to 4.1% for the WT cells (Fig. 5*E*). In fact, 67.9 and 79.1% of elongated *dnaX*:*S617P* and *dnaX*:*L635P/D636G* cells, respectively, were BrdU-positive.

### dnaX:mut strains have significantly more cell death

**Figure 6:**
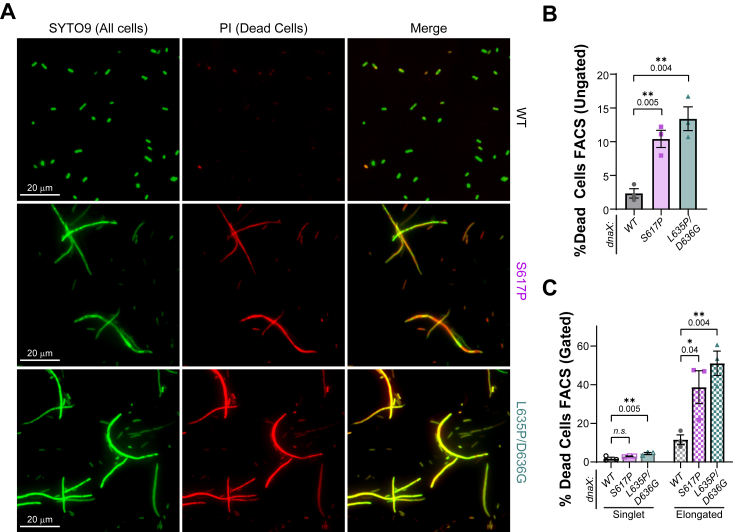
**Increased cell death in *dnaX*:*mut* strains.***A*, *dnaX*:*mut* strains grown in Miller LB from a late exponential population were stained for SYTO9 (all cells) and PI (dead cells) and imaged by epifluorescence microscopy. The same populations were analyzed by FACS (n = 5000 events, each for three biological replicates) and the percentage of dead cells (stained with propidium iodide) from the (*B*) ungated population and (*C*) the gated singlet and elongated subpopulations was quantified and plotted. Error bars represent the SE and are contained within the symbol if not visible. *Black* bars indicate statistically significant differences where *p-*values are indicated and represented by ∗<0.05 and ∗∗<0.001 from an unpaired two-sided *t* test.

To investigate whether significant cell death in the *dnaX*:*mut* strains, likely from overwhelming single-strand DNA gaps and SOS, is contributing to measured slower growth rates, flow cytometry and microscopy were utilized to quantify populations of live and dead cells stained with SYTO9 and propidium iodide (PI) in late exponential stage (Fig. 6*A*). The percentage of dead cells measured by flow cytometry in S617P (10.4%) or *dnaX*:*L635P/D636G* (13.4%) were significantly higher than WT (2.3%) (Figs. 6*B* and S8). Interestingly, both *dnaX*:*S617P* and *dnaX*:*L635P/D636G* exhibited a significant proportion of elongated cells that were PI-positive (Fig. S8), which were also evident in the microscopy images (Fig. 6*A*). The more intense red color observed in the elongated cells is reflected in the flow cytometry data, where 38.7% and 51.0% of cells gated outside of the singlet population in *dnaX*:*S617P* or *L635P/D636G* were not viable (Fig. 6*C*).

### CLC stimulates DnaB unwinding *in vitro*

Examining the impact of perturbed τ-α interactions *in vivo* gave a more global representation of the negative effect that disrupting coupling had on gross genomic stability. However, to obtain a mechanistic explanation for the importance of τ-α interactions, purified τ_3_-CLC mutants were used in *in vitro* assays. In addition to S617P and L635P/D636G, a truncated τ_3_(I618X)-CLC was purified as a control that deletes the last 25 amino acids of the C-terminus that primarily interacts with α subunit (Fig. S9*A*).

**Figure 7:**
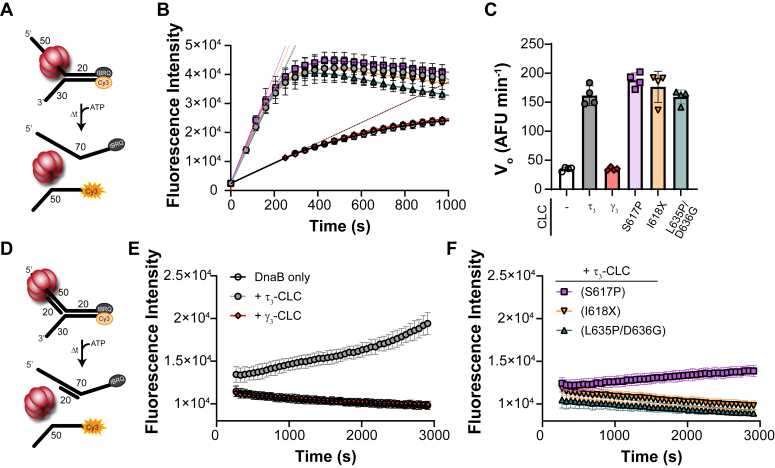
**DnaB unwinding activity is stimulated by the presence of τ-CLC *in vitro*.***A*, a Cy3-BHQ forked DNA substrate with a 5′ overhang for DnaB helicase loading is unwound upon addition of ATP by (*B*) monitoring the averaged Cy3 signal over time. *C*, the maximal rate (*V*_*0*_) of the fluorescence increase were plotted (n = 8) for the respective CLC proteins. *D*, a duplex translocation assay where a 20-base pair duplex was added to the 5′ arm and unwinding is monitored by a sensitization of the Cy3 signal over time (n = 4) for (*E*) DnaB only (○ *black*), +τ_3_-CLC (• *gray*), and γ_3_-CLC (◆ *red*) or (*F*) DnaB plus S617P (▪ *lavender*), I618X (▼ *yellow*), or L635P/D636G (▲ *teal*). Error bars represent the SD.

The sequence shared by both τ and γ proteins enables any of the associated CLCs to load the β clamp on DNA in an ATP-dependent manner (36, 37). Therefore, to verify that the activity of the purified mutated CLC proteins are not affected, a NADH-coupled ATPase clamp-loading assay was performed. As expected, all mutant CLC proteins including γ_3_-CLC showed similar ATPase activity, verifying that the CLC proteins were active (Fig. S9*B*). τ–DnaB interactions are also necessary to maintain a stable but dynamic replisome necessary for efficient fork progression connected through domain IV, which is absent in γ_3_-CLC but present in all other τ_3_-CLC mutants (Fig. 1) (5, 11). A fluorescent DNA unwinding assay using a quenched forked substrate in the presence of DnaB helicase and the mutant CLC proteins was performed to analyze any stimulation of unwinding activity upon interaction with τ_3_-CLC (Fig. 7*A*). Both DnaB on its own or in the presence of γ_3_-CLC had low unwinding rates; however, inclusion of other mutant τ_3_-CLC proteins all stimulated DnaB unwinding similar to that for WT τ_3_-CLC (Fig. 7, *B* and *C*).

Like the duplex unwinding assay, the same BHQ-Cy3 forked substrate was utilized with the addition of a short 20 mer strand that anneals to the 5′ arm to test the duplex translocation ability of DnaB prior to unwinding (Fig. 7*D*). DnaB on its own is unable to effectively translocate over this duplex region and unwind the fork; however, the addition of τ_3_-CLC helps to dynamically alter the DnaB hexamer conformation towards a dilated state, allowing duplex translocation followed by effective fork unwinding (Fig. 7*E*). Domains IV and V of τ_3_-CLC are also important for inducing a dilated state in DnaB as γ_3_-CLC is unable to facilitate duplex translocation. Like γ_3_-CLC, τ_3_(I618X)-CLC and τ_3_(L635P/D636G)-CLC are unable to induce the dilation of DnaB for duplex translocation. However, τ_3_(S617P)-CLC does allow for about one-third of the rate of DnaB dilation/translocation compared to WT (Fig. 7*F*). Therefore, structural features within domain V of τ propagate through to domain IV to impact the DnaB hexamer conformation for effective duplex translocation, and these τ mutants have a reduced ability to alter the DnaB conformation.

### Leading strand synthesis decouples when τ–Pol α interactions are fully disrupted

**Figure 8:**
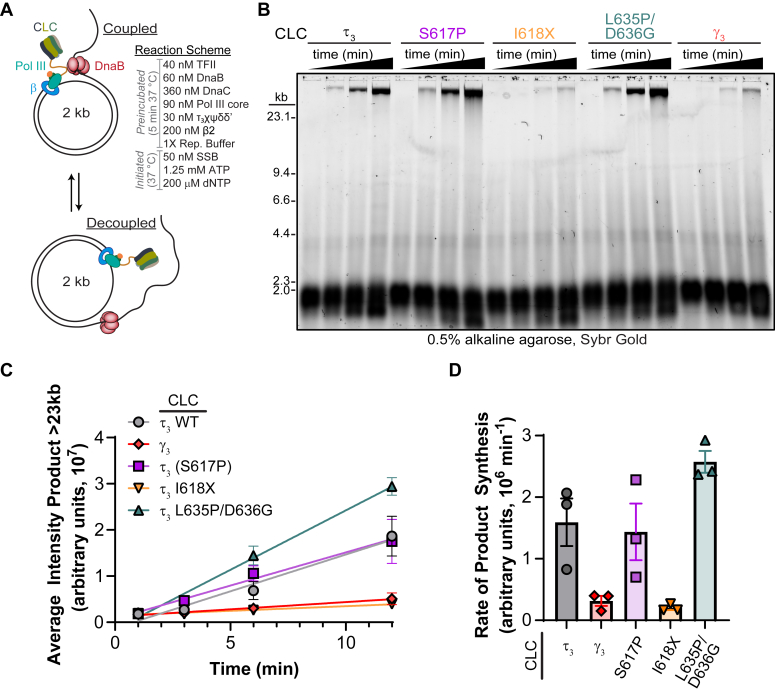
**Only deletion of the extreme C terminus of τ disrupts replisome coupling *in vitro*.***A*, an *E. coli* replisome was reconstituted *in vitro* on a tailed form II DNA substrate and (*B*) the nascent leading strand-synthesized products at different timepoints were visualized on a 0.5% alkaline agarose gel using SYBR Gold. *C*, the average intensity of the total product was plotted against time (n = 3 technical replicates). (WT - ○ *gray*, Gamma - CLC - ◆ *red*, S617P - ▪ *lavender*, I618X – ▼ *yellow*, L635P/D636G - ▲ *teal*). *D*, a bar graph depicts the average rate of total product synthesized in the presence of the respective CLCs with error bars representing SE.

To directly test whether disruption in replisome coupling results from mutations of the τ-α interface, mutant τ_3_-CLC proteins were utilized in a reconstituted *in vitro* leading strand replisome assay (Fig. 8*A*). Previously it was shown that the τ-mediated physical interaction between helicase and polymerase is required for the high rate of replication fork progression (5). The tailed form II rolling circle substrate is a nicked circular DNA template with a 5′ overhang that mimics an open replication fork, making it an ideal substrate to study DNA replication *in vitro* (38). Utilization of γ_3_-CLC or τ_3_(I618X)–CLC complexes in this rolling circle assay resulted in a significant reduction in overall leading strand product compared to a WT τ_3_–CLC complex (Fig. 8, *B* and *C*). Interestingly, both τ_3_(S617P)-CLC and τ_3_(L635P/D636G)-CLC showed similar (if not slightly elevated) leading strand products compared to WT. It appears that only complete disruption of the τ-α interface through deletion of the last 25 amino acids (*i.e.* I618X) fully inhibits the leading strand replisome, while moderate perturbation at the interface (S617P or L635P/D636G) are not sufficient to disrupt coupling in this ideal *in vitro* system where DnaB dilation is generally unnecessary.

## Discussion

The model of a stable replisome and uninterrupted DNA replication process from origin to terminus has been recently challenged by the discoveries of more dynamic replisome components (10, 39). Even so, the DnaB helicase has the longest dwell time bound to DNA in comparison to the other replisome proteins and is believed to act as the stable platform upon which the rest of the components reassemble (6). The Pol III∗ subassembly (αεθ-τ_3_δδ’, without the β-clamp) is frequently exchanged with Pol III∗ in solution, and for this exchange to occur, at least one τ-subunit should be present in the CLC (6, 7, 40). Furthermore, the rate of DNA synthesis is kinetically discontinuous with frequent pausing, during which DNA unwinding slows down to allow recoupling with the polymerase (9). Therefore, by altering the speed of helicase unwinding, kinetic coupling can be maintained through physical coupling with the polymerase to limit the production of ssDNA gaps (9, 27). With Pol III∗ being constantly exchanged in a concentration-dependent manner, it is important to maintain and regulate a physical link to the more stable helicase hub through τ to couple unwinding with synthesis.

Previous studies have shown that the C-terminus of τ consists of a structured region within domain V (residues 507–617, H6 in Fig. 1*C*) but is unstructured beyond S617 unless it is bound to α (modeled H7 in Fig. 1*C*) (15, 16). S617 is located near the end of the first α-helix (H6) that interacts directly with the α-subunit of Pol III, and substitution of Ser to Pro would likely disrupt the entire H6 and/or destabilize binding of the downstream-induced β-turn-H7 reducing affinity with α. The induced H7 α-helix spans from N622 to D636 and is modeled to interact within a hydrophobic patch on α (15). In addition to S617P, we chose to either terminate τ at I618X or combine mutations (L635P and D636G) in the induced H7 to better understand how interactions between α and τ through H6 and H7 couple and regulate DNA synthesis and unwinding to prevent genomic instability during replication.

The parental strain MG1655 was genetically engineered to introduce targeted mutations in the *dnaX* gene corresponding to S617P and L635P/D636G mutations in the τ protein, to perturb (but not eliminate) Pol III α-τ interactions occurring at the C-terminus of τ. Although we made several attempts to edit *dnaX:I618X*, we could never recover any successful clones, highlighting the importance of the downstream H6 and H7 interactions with α required for survival. Previously, deletion of the last 7 (Δ636–643) or 11 (Δ632–643) residues moderately or severely inhibited the interaction with α, even though these residues are past H6 (15). The two successfully edited *dnaX*:*mut* strains both showed reduced growth rates, fitness, and survival rates in addition to increased levels of SOS and mutagenesis. Reduced growth rates and fitness in both mutant strains indicate that by altering the α-τ interaction, there are adverse impacts on the continuity of DNA replication, cell segregation, and strain survival.

*dnaX*:*mut* strains displayed altered cell morphologies and increased odd numbers of chromosomes corresponding to asynchronous replication. Elongated cellular filamentation is a phenotypic indication of cellular stress (41), and asynchronous replication can generate complex genomic intermediates and stretches of unreplicated DNA. In defense, the SOS response will be upregulated to inhibit cell division by expression of *sulA* to allow for the resolution of any unreplicated DNA through increased expression of DNA repair genes, including the translesion polymerase, Pol V (42, 43). It was evident that these *dnaX*:*mut* strains had higher levels of ssDNA gaps as detected by PLUG, confirming that targeted decoupling leads to abundant ssDNA gaps. Those ssDNA gaps activated the SOS response in an attempt at survival but also resulted in increased chromosomal complexities, indicative of severe genomic damage and explaining the severely elongated cellular phenotypes. Cellular elongation generally persisted into stationary phase in these strains, consistent with a slow replication/repair phenotype and also reflecting severe and adverse genomic complications. *dnaX*:*mut* strains also had reduced survival rates, with a larger proportion of nonviable cells in the elongated subpopulations. Therefore, this establishes a pattern whereby persistently disrupted replisomal coupling leads to accumulation of ssDNA gaps, resulting in SOS induction and cellular filamentation ultimately overwhelming the genomic repair process and ending in cell death.

We expected that by altering the τ-α interactions, replisome coupling between unwinding and synthesis will be primarily affected, resulting in these genomic and cellular markers of stress, but τ also interacts with DnaB (primarily through Domain IV). This τ–DnaB interaction can also affect the speed of helicase in controlling replisome progression, where τ can bring about a conformational change in DnaB that allows it to unwind faster (5, 44). Interestingly, while WT and mutant τ_3_CLC proteins were all capable of stimulating DnaB unwinding to varying degrees, only WT CLC was able to effectively allow a conformational change in DnaB to dilate and translocate over dsDNA. This constricted-dilated DnaB regulation may be one of the most important factors for maintaining an effective and dynamic replisome (27).

Interestingly, τ_3_-CLC(L635P/D636G) was capable of both stimulating DnaB unwinding and synthesizing leading strand products at levels even in excess of WT but did not allow for effective DnaB dilation over duplex DNA. τ_3_-CLC(S617P) had a much-reduced ability to dilate DnaB, likely explaining why *dnaX*:*S617P* exhibited less extreme phenotypes *in vivo* compared with *dnaX*:*L635P/D636G*. *dnaX*:*L635P/D636G* exhibits the greatest frequency of dead cells, mutations, SOS induction, and single strand gaps, leading to the most significant cellular elongation and slower growth phenotypes. We suspect that the extreme C-terminus of τ not only acts as a primary interaction site with α but also as a regulation switch to help with Pol III∗ recycling, directly impacting DnaB unwinding regulation. In the absence of this regulation switch, Pol III∗ is not effectively recruited or evicted during rounds of lagging strand synthesis or leading strand blocks. For *dnaX*:*L635P/D636G*, this τ-CLC mutant stimulates the unwinding of DnaB but is unable to regulate the DnaB conformation to control the speed when Pol III∗ is needed or to overcome genomic obstacles *in vivo*, leading to increased ssDNA gaps and rampant cellular stress.

Replisome decoupling may occur because of several genomic challenges, disrupted replisome interactions, or the inability to induce conformational switching to control the kinetics as described previously (45). During decoupling, polymerase progression may be inhibited while releasing the helicase to continue unwinding, leaving regions of ssDNA exposed. Accumulation of excess ssDNA is a substrate for RecA binding and filamentation, initiating the SOS response to induce gene expression of DNA repair genes that contribute to error prone DNA synthesis for survival (43, 46). τ acts as the lynchpin to physically connect the helicase and polymerase and is shown to play a multifaceted role in ensuring that coupling extends far beyond a mere physical connection but also impacting conformation. By regulating a transition between constricted and dilated states in DnaB, τ can control the unwinding speed to ensure the helicase and polymerase remain coupled despite constant polymerase recycling and differential enzymatic rates to maintain efficient replication and ensure genomic integrity.

## Experimental procedures

### Cloning and expression of τ-CLC mutants

Mutations in the τ protein were introduced using primers containing the targeted mutation and novel restriction site (Table S3) in PCR reactions with pCOLADuet-1 containing the *dnaX* gene (47) as the template (gift from Linda Bloom) and Platinum SuperFi PCR Master Mix (Thermo Fisher Scientific). Colonies were PCR screened and confirmed by restriction digested and whole plasmid sequencing (Plasmidsaurus). Confirmed mutant plasmids were transformed into Rosetta2 expression cell line, and overexpression of τ_3_δδ’ψχ was induced with IPTG (1 mM). Purification of the CLC complexes were performed as previously described (47).

### Bacterial CRISPR-Cas9 genome editing

The parental *E. coli* strain, MG1655, was used to create genetically edited CRISPR/Cas9 mutants of *dnaX* gene using a dual vector targeting system. Synthetic 40 base pair oligonucleotides with sequences homologous to *dnaX* targeting region acting as gRNA (Table S3) were ligated into the pCRISPR plasmid (Addgene: 42875) (48). First, pREDCas9 (Addgene: 71541) (49) was electroporated into MG1655 and plated on 100 μg/ml spectinomycin (Gold Biotechnology Inc) LB agar plates (10 g/L tryptone, 5 g/L yeast extract, 10 g/L NaCl, and 15 g/L agar) at 30 °C. Then, both 100 ng of pCRISPR-g*dnaX* and 1 μM of ssDNA oligonucleotides containing homologous region to *dnaX*, targeted single point mutations, and novel restriction sites for screening were simultaneously electroporated and plated on spectinomycin/kanamycin (50 μg/ml) LB agar plates and incubated at 30 °C. Colony PCR followed by restriction digest was used to verify the presence of the novel restriction site (Fig. S2), ensuring successful genomic editing. These *dnaX*:*muts* were then allowed to grow at 42 °C in LB/Kan to cure the pREDCas9 plasmid from a temperature sensitive origin, p15A. The genomes of *dnaX*:*S617P* and *dnaX*:*L635P/L636G* were sequenced (Plasmidsaurus) to further verify the single point mutations in the genome at the targeted site and eliminate the possibility of off-target mutations (Table S4). All further experiments were grown at 37 °C, unless specified otherwise.

### τ–α pull down assay

Pull-down assays were performed by adding 50 μl of nickel resin (HisPur Ni-NTA Resin, Thermo Fisher Scientific) to a 1.5 ml Eppendorf tube and spun down at 800*g* for 2 min. The supernatant was removed, and the pellet was resuspended in 50 μl of binding buffer [20 mM sodium phosphate, 0.125 M NaCl, 25 mM imidazole, 1 mM ATP, pH 7.4], spun down again, and the supernatant discarded. A 1:1 ratio of His-tagged Pol α to τ_3_-CLC are mixed with the resin and allowed to incubate at 4 °C for 30 min before spinning down and transferring the flow through solution to a new tube. The pellet was resuspended in 50 μl of binding buffer, spun down, and transferred to a new tube as the first wash, taking care not to disturb the resin pellet. This wash step is repeated 12 more times (13 total) to ensure that all unbound protein is removed before resuspending the resin in 50 μl of elution buffer [20 mM sodium phosphate, 0.125 M NaCl, 500 mM imidazole, pH 7.4], spinning down, and transferring the elution solution to a new tube. Finally, the flow through, washes, and elution are electrophoresed on a 10% SDS Bis-Tris acrylamide gel, stained with Simplyblue Safestain (Thermo Fisher Scientific), and imaged on a Gel DocTM EZ system (Bio-Rad).The bands corresponding to τ and α were quantified separately using ImageQuant (v.10.2), and the fractional saturation was calculated by obtaining the band intensity ratio of τ (or γ) to α.

### Growth assay

Overnight cultures were diluted to reach an *A*_*600*_ value ∼0.01 and 200 μl of diluted culture was aliquoted into white, clear bottomed 96-well plates, and analyzed on a Tecan Spark microplate reader. The absorbance values were recorded at 30-min intervals over 24 h and the temperature was maintained at 37 °C with aeration at 240 rpm to control for regular growth profiles. Data was analyzed using GraphPad Prism (v10.2) and fit into a modified 4-parameter Gompertz growth model as follows:

(1) $$w\left(t\right)=B+A^{{-e}^{\left(\frac{k_{g}\times 2.7182}{A}\times \left(T_{lag}-t\right)+1\right)}}$$

where *w(t)* is the density as function of time, *B* is the lower asymptote, *A* is the higher asymptote, *T*_*lag*_ is the lag time of the culture, *t* is time, and *k*_*g*_ is growth rate coefficient. The absolute growth rate (*k*_*z*_) can be calculated using the following equation:

(2) $$k_{z}=\frac{k_{g}\times 2.7182}{A}$$

The absolute growth rates were plotted and analyzed for any significant differences using unpaired two-tailed *t* test with GraphPad Prism (v10.2).

### Live/dead analysis

To visualize and measure the viability of *dnaX:mut* strains, the LIVE/DEAD *Bac*Light Bacterial Viability Kit L7012 (Thermo Fisher Scientific) was used. Overnight grown cultures were diluted 100-fold in fresh Miller LB media and allowed to grow till *A*_*600*_ reached late exponential phase (0.8∼0.9). Twenty five milliliters of culture was centrifuged at 4000*g* for 20 min and the pellets were resuspended with 2 ml of 0.85% sterile NaCl solution. One milliliter of resuspended solution was added to 20 ml of 0.85% NaCl solution (70% ethanol for dead cells as a control) and incubated at room temperature for 1 h with mixing at 15-min intervals. The samples were then centrifuged at 4000*g* for 20 min and resuspended in 20 ml of 0.85% NaCl and centrifuge step was repeated. The pellets were resuspended in 10 ml of 0.85% NaCl, and 3 μl of 1:1 mixture of SYTO9 (3.34 mM) and PI (20 mM) was added to 1 ml of resuspended bacterial suspension and incubated in the dark for 15 min.

For microscopy, 5 μl of stained solution was added to the slide followed by 3 μl of included mounting media, coverslip added, and sealed with clear nail polish. Microscopy images were visualized using Olympus Brightfield microscope IX-81 (Olympus Corp) with a 60× objective lens upon oil immersion. Images were visualized with FITC filter for SYTO9 and TRITC filter for PI. The stained bacterial suspensions were used for analysis on the flow cytometer (Cytek Northern Lights flow cytometer).

### Competition assay

A derivative of MG1655, EAW214, containing an *araBAD* mutation within the promoter region was used as the control strain for this assay (gift from Mike Cox) (28). When plated on tetrazolium arabinose (TA) plates, the *ara*^*-*^ strains appear as red colonies as they cannot utilize arabinose as carbon source. TA plates contain 1% arabinose (Oakwood products) and 0.2 mg/ml triphenyl tetrazolium chloride (Sigma). The *dnaX:WT* and EAW214 strains were mixed with equal numbers from an overnight culture. The *dnaX:S617P* and EAW214 strains were mixed in a 3:1 ratio, and *dnaX:L635P/D636G* and EAW214 were mixed in a 4:1 ratio and diluted 10^−6^ in sterile water and plated on TA plates to ensure a 50:50 ratio of red:white colonies on day 0. The mixed strains were diluted 100-fold in LB media and allowed to grow for 24 h, diluted, and plated on TA plates. This procedure was repeated until there was a significant drop in one population of cells with respect to the competing population (27, 28). The selection rate of each of the strains was calculated using the following equation:

(3) $$selection\;rate=r=\ln\frac{A_{1}}{A_{0}}-\ln\frac{B_{1}}{B_{0}\;}$$

where *A*_*0*_ and *B*_*0*_ are colony forming units (CFU) fractions of strains A and B at time 0 and *A*_*1*_ and *B*_*1*_ are CFU fractions of strains A and B after 24 h. The selection rates were plotted and analyzed for significance using unpaired two-tailed *t* test with GraphPad Prism (v10.2).

### Microscopy

To examine exponential phase cells, overnight cultures were diluted 1000-fold and grown until *A*_*600*_ reached ∼0.4 before harvesting. Stationary phase cells were grown overnight. 1 ml of culture was pelleted, washed with PBS, and resuspended in 70% ethanol. 2 ml of the fixed cell samples followed by 3 μl of staining and mounting solution containing DAPI (Thermo Fisher Scientific), DABCO, glycerol, and PBS were spotted onto microscope slides, covered with a coverslip, and sealed with clear nail polish. Microscopy images were visualized using an Olympus Brightfield Microscope IX-81 (Olympus Corp), with a 60× objective lens upon oil immersion. Cell lengths were measured using cellSens software (Olympus). The cell lengths were plotted and analyzed for significance using a Mann-Whitney two-sided U-test with GraphPad Prism (v10.2).

### Chromosome content

Overnight grown cultures were diluted 1000-fold and allowed to reach an *A*_*600*_ value of ∼ 0.35 prior to treating with rifampicin (300 μg/ml) and cephalexin (30 μg/ml) and then allowed to grow out for 4 h at 37 °C to complete chromosome synthesis (27). Cells were pelleted, washed in 1× TE buffer, and fixed in 70% ethanol. Fixed cells were pelleted, washed, and resuspended in PBS containing 1.5 μM of Sytox Green (Invitrogen) and allowed to stain for 30 min in the dark at room temperature. The stained samples were diluted with sheath fluid and analyzed by flow cytometry (BD FACSVerse flow cytometer, BD Biosciences). Unstained cell samples were used as a control to set the gating. Data was plotted using FlowJo software (BD Biosciences). A temperature-sensitive *dnaA(ts)* strain, CM742, carrying the *dna46* mutation was prepared as previously described (27).

### SOS induction

The plasmid, pEAW915, contains the SuperGlo GFP gene under the control of the *recN* promoter (gift from Mike Cox); a gene that is induced in the early phase of the SOS response (32). This reporter plasmid was transformed into *dnaX*:*mut* strains by electroporation. Overnight grown cultures were diluted 100-fold in LB media or LB media with 0.001 μg/ml mitomycin C (Thermo Fisher Scientific) and 200 μl aliquots were added into white, clear bottomed 96-well plates. The cultures were incubated at 32 °C (to maintain normal growth profiles) and the absorbance (*A*_*600*_) and the fluorescence (ex 474 nm/em 509 nm) were read at 30-min intervals over a course of 24 h using Varioskan LUX microplate reader (Thermo Fisher Scientific). For analysis, specific fluorescence was calculated using the following equation (32).

(4) $$Specific\;fluorescence=\frac{Fluorescence\;}{{Absorbance\;(A}_{600})}$$

And the maximum fluorescence at equivalent times were analyzed for significant induction of SOS using a unpaired two-tailed *t* test with GraphPad Prism (v10.2).

To analyze the cells using flow cytometry, 200 μl of overnight grown culture (stationary phase) was pelleted and washed thrice with 1× sterile PBS. The cells were resuspended in 1 ml of 1× PBS and analyzed using the flow cytometer (Cytek Northern Lights flow cytometer). For microscopy, cells were prepared using the same procedure as for flow cytometry, with an additional step of fixing the cells with 4% paraformaldehyde in PBS and incubated at room temperature for 20 min, followed by washing thrice with 1× PBS and resuspended in 500 μl of 1× PBS. Four microliters of the bacterial suspension was spotted on a slide and slides were prepared as mentioned previously. Slides containing SuperGlo GFP–transformed cells were visualized using an Olympus Brightfield Microscope IX-81 (Olympus Corp), with a 60× objective lens upon oil immersion.

### Rifampicin mutagenesis assay

Overnight grown cultures were diluted 1000-fold and allowed to grow for 24 h before rediluting and growing for an additional 24 h. 100 μl of aliquots were plated on LB agar plates containing rifampicin (50 μg/ml) directly or serially diluted and plated on LB agar plates (50, 51). The rate of mutagenesis was calculated using the following equation:

(5) $$mutation\;frequency=\frac{A}{B\times 10^{6}}$$

where *A* is the number of CFU on plates with rifampicin and *B* is the number of CFU on plates without rifampicin. The growth rates were plotted and analyzed for significance using unpaired two-tailed *t* test with GraphPad Prism (v10.2).

### Immunofluorescence assay

Overnight grown cultures were diluted 1000-fold and allowed to reach an *A*_*600*_ value of ∼0.5, pelleted at 4000*g* for 15 min, the pellet was washed thrice with PBS, fixed with 1 ml of 4% paraformaldehyde in PBS, and incubated at room temperature for 20 min. Following fixing, cells were pelleted, washed twice with PBS, and the cell pellets were resuspended in 70% cold ethanol and stored at 4 °C until ready for immunolabeling. For the PLUG labeling assay, the cells stored in 70% ethanol was pelleted, washed with PBS, and resuspended in 100 μl of reaction buffer (5 U of Klenow fragment [NEB], 1X NEBuffer 2, 10 μM each of dATP, dCTP, dGTP, and BrdU [Thermo Fisher Scientific]), and incubated at 37 °C for 30 min. The cell pellets were resuspended and washed twice in PBS, resuspended again in 100 μl of blocking solution of 4% BSA in 1X TBST [50 mM Tris and 150 mM NaCl, 0.1% Tween, pH 7.6], and incubated at 4 °C overnight on a rocker. The cells were pelleted and then resuspended in 100 μl of primary antibody solution with 1:100 mouse anti-BrdU (BD Biosciences) in 2% BSA in TBST and incubated at room temperature on a rocker for 1.5 h. The cells were then pelleted and washed thrice with TBST and resuspended in 100 μl of secondary antibody solution with 1:500 goat anti-mouse IgG (H + L) AF647 (SouthernBiotech) and incubated at room temperature on a rocker for 1.5 h. The cells were pelleted, washed with TBST, and resuspended in 1 ml of PBS.

To prepare the cells for microscopy imaging, 2 μl of the cells were spotted on clean microscope slides, and once dried, 2 μl of SlowFade Diamond Antifade Mountant with DAPI (Thermo Fisher Scientific) was added, covered immediately with a coverslip, and sealed with clear nail polish. The slides were visualized using an Olympus Brightfield Microscope IX-81 (Olympus Corp), with a 60× objective lens upon oil immersion. Both DAPI filters and Cy5 filters were used for visualization.

To analyze the fluorescence intensities, cells were diluted with sheath fluid and analyzed using flow cytometry (BD FACSVerse flow cytometer, BD Biosciences). Fixed cells that were unlabeled were used as a gating control to test for BrdU negative cells. Data was plotted using FlowJo software (BD Biosciences).

### Leading strand synthesis assay

*In vitro* leading stand DNA replication assays were performed with a reconstituted *E. coli* replisome (23) using the tailed-form II substrate for rolling circle amplification created from the vector pSCW01 as described (38). The fork oligonucleotide Cy5-DNA200 (IDT) (Table S3) was annealed to the gapped plasmid. DnaB and DnaC were purified as described (27, 52). *E. coli* SSB, β-clamp, and γ_3_δδ’ψχ were generous gifts from Charles McHenry. Pol III core was purified as described (53).

For the replication assay, 40 nM DNA template was incubated with 30 nM τ_3_δδ’ψχ, 90 nM Pol III core (α,ε, and θ), 60 nM DnaB (monomer), 360 nM DnaC, and 200 nM β-clamp at 37 °C for 5 min in replication buffer (50 mM Tris. HCl pH 7.5, 10 mM MgCl_2_, 10 mM (NH_4_)_2_SO_4_, and 4 mM DTT) and initiated with 1.25 mM ATP, 50 nM SSB, and 200 μM dNTPs. The reactions were terminated with 1.5 μl of 0.5 M EDTA and 3 μl of DNA loading dye (6 mM EDTA, 300 mM NaOH, 0.25% [w/v] bromocresol green, 0.25% [w/v] xylene cyanol FF, 30% glycerol) after reaching their respective timepoints (38). Reactions were electrophoresed on 0.5% alkaline agarose gels in 1× alkaline agarose buffer at 15 V for ∼15.5 h. Gels were neutralized in 2× TAE for 2 h on a rocker, stained with 1× SYBR Gold (Invitrogen) in 2× TAE for 2 h, and imaged using GE Typhoon FLA 9000 (Cytiva) for fluorescence. The bands in the gels were quantified using ImageQuant (v.10.2).

### NADH-coupled ATPase assay

To verify the activity of mutant τ_3_-CLC proteins, a NADH-coupled ATPase assay was performed. The master mix was prepared with 1× reaction buffer (20 mM Hepes–KOH, 5 mM Mg(OAc)_2_, 50 mM potassium glutamate, 5% glycerol, 0.2 mg/ml BSA, 4 mM DTT, pH 7.5), 2 mM ATP, 8 mM phosphoenol pyruvate, 480 nM NADH, 1 μM ssDNA DNA165, and 3.6 μl of pyruvate kinase/lactate dehydrogenase enzyme (Sigma). The samples were aliquoted in to a 384-well black microplate (Corning) and initiated with 250 nM of β-clamp and 250 nM of the respective CLC protein. The fluorescence (ex 340 nm/em 460 nm) was read at 30-s intervals over a course of 25 min on a Tecan microplate reader. The fluorescence values were plotted as a function of time and the steepest gradient values were plotted on GraphPad Prism (10.2).

### DNA unwinding and translocation

The forked DNA substrate for unwinding was prepared by annealing Cy3-DNA165 with DNA181-BHQ in a 1:1.1 ratio in 1× annealing buffer (20 mM Tris–HCl, 4% glycerol, 0.1 mM EDTA, 40 μg/ml BSA, 10 mM DTT, 10 mM Mg(OAc)_2_ (pH 8)) and incubated at 37 °C overnight. The unwinding reactions contained 1× reaction buffer (20 mM Hepes–KOH, 5 mM Mg(OAc)_2_, 50 mM potassium glutamate, 5% glycerol, 0.2 mg/ml BSA, 4 mM DTT, pH 7.5), 100 nM of DnaB, and 100 nM of respective CLC protein and incubated on ice for 10 min, followed by the addition of 50 nM of annealed DNA substrate and incubated further for 10 min. The samples were then aliquoted in to a 384-well black microplate (Corning), initiated with 1 mM ATP and 100 nM unlabeled DNA165 (as a trap), and fluorescence (ex 510 nm/em 580 nm) was read at 30-s intervals over a course of 25 min on a Tecan microplate reader. DNA unwinding was monitored through an increase in fluorescence upon release of the BHQ substrate, and the maximal slope was used to calculate the initial velocity (V_0_).

The forked DNA substrate for translocation was prepared by mixing Cy3-DNA165, DNA181-BHQ, and DNA180 in 1:5:1 ratio respectively and annealed as mentioned previously. The methodology for translocation was the same as for unwinding with only difference being that 50 nM of annealed DNA translocation fork substrate was added in the place of DNA unwinding fork substrate.

## Data availability

All biochemical data presented in this study including, value, gels, images, datasets as well as any strains or plasmids are available upon request to the corresponding author.

## Supporting information

This article contains supporting information (27, 28, 32, 38, 47, 53, 54).

## Conflict of interest

The authors declare that they have no conflicts of interest with the contents of this article.
