# Supplementary material for: Tau-mediated coupling between Pol III synthesis and DnaB helicase unwinding helps maintain genomic stability
Source: J Biol Chem. 2024 Aug 29;300(10):107726. doi: 10.1016/j.jbc.2024.107726 (PMC11470591; doi:10.1016/j.jbc.2024.107726)
Supplement: Supporting Figures and Tables [file mmc1.pdf]

SUPPORTING FIGURES AND TABLES

Tau mediated coupling between Pol III synthesis and DnaB helicase unwinding helps maintain genomic stability

Malisha U. Welikala[1], Lauren, J. Butterworth[1], Megan S. Behrmann[1,†], and Michael A. Trakselis[1*]

[1]Department of Chemistry and Biochemistry, Baylor University, Waco, Texas, 76798, USA
[†]Current Address: National Cancer Institute, Bethesda, MD, 20814, USA.
*To whom correspondence should be addressed: *Michael A. Trakselis, One Bear Place #97348, Waco, TX 76798-7348. Tel 254-710-2581; Email: michael_trakselis@baylor.edu

Supplementary Table S1: Strains

Strains	Genotypes	Description
MG1655	*E. coli* K-12	Wild type parental strain, CGSC# 7740
EAW214	MG1655 ΔaraBAD	Deletion of araBAD promoter (1)
CM742	*dnaA(ts) dna46*	Contains a temperature sensitive *dnaA* mutation, CGSC# 12549
MUW1	MG1655: *dnaX*:S617P	Contains a *dnaX* point mutation
MUW2	MG1655: *dnaX*:L635P/D636G	Contains a *dnaX* point mutation

Supplementary Table S2: Plasmids

Plasmids	Source	Description
pEAW915	(2)	SuperGLo GFP plasmid with the *recN* promoter
pSCW01	(3)	TFII substrate
pET16b-Pol III core	(4)	Overexpression of Pol III core (αεθ)
pET11b DnaB	(5)	Overexpression of DnaB and mutants
pETDuet-1-*holB/C/D*	(6)	Overexpression of delta', chi, and psi subunits
pCOLADuet-1-*dnaX holA*	(6)	Overexpression of delta and τ_3-CLC and mutants
pET28b-DnaC	(7)	Overexpression of DnaC

Supplementary Table S3: Oligonucleotides

Name	Sequence (5'-3')
CRISPR S617P FW	5'AAACCGAAGAAAAACTTGCGCAGGCGCGCGAGTCCATTATTGG
CRISPR S617P RV	5'AAAACCAATAATGGACTCGCGCGCCTGCGCAAGTTTTTCTTCG
CRISPR L635P/D636G FW	5'AAACTAATATTCAGACCCTGCGTCGGTTCTTCGATGCGGAGCG
CRISPR L635P/D636G RV	5'AAAACGCTCCGCATCGAAGAACCGACGCAGGGTCTGAATATTA
S617P HR oligo	5'CGAAGAAAAACTTGCGCAGGCTCGAGAGCCAATTATTGCCGATAATAATATTCAGACCCT
L635P/D636G HR oligo	5'GACCCTGCGTCGGTTCTTCGATGCGGAGCCCGGGGAAGAAAGTATCCGCCCCATTTGATC
SDM S617P FW	5'CAGGCTCGAGAGCCAATTATTGCGGATAATAATATTC
SDM S617P RV	5'CAATAATTGGCTCTCGAGCCTGCGCAAGTTTTTC
SDM L635P/D636G FW	5'GATGCGGAGCCCGGGGAAGAAAGTATC
SDM L635P/D636G RV	5'GAAGAACCGACGCAGGGTCTGAATATTATTATC
SDM I618X FW	5'CGCAGGCTCGAGAGTCCTGAATTGCGGATA
SDM I618X RV	5'CAAGTTTTTCTTCGTATATCGCCTGACGCC
DNA165	5'TCCCACCCAACCCGACCGGCATCTAGTCTGGTAGCGTGAGCGAACGGACC
Cy3-DNA165	5'<u>3</u>TCCCACCCAACCCGACCGGCATCTAGTCTGGTAGCGTGAGCGAACGGACC
DNA180	5'TGACGTCGCACACCGTGCTC
DNA181-iBHQ	5'TTTTTTTTTTTTTTTTTTTTTTTTTTTTTTGAGCACGGTGTGCGACGTCAGCCGGTCGGGTTGGGTGGGA<u>Q</u>
DNA197	5'ATTTGACTCC
DNA198	5'CATGGACTCGCTGCAG
DNA199	5'GAATGACTCGG
Cy5-DNA200	5'<u>5</u>AAAAAAAAAAAAAAAAGAGTACTGTACGATCTAGCATCAATCACAGGGTCAGGTTCGTTTGGGAGTCAAAT

<u>3</u>-Cy3,<u>5</u>-Cy5, <u>Q</u> - Black hole quencher

Supplementary Table S4: Whole Genome Sequencing Results

			Genome		aa			
Strain:	POS	TYPE	REF	ALT	REF	ALT	aaPOS	Gene Name
MG1655:	1890031	snp	G	A	G	D	25	manganese efflux pump MntP
dnaX:S617P	3077921	snp	C	A	I	L	54	Putative hydratase
	3103780	snp	A	C	n/a	n/a	n/a	tRNA-Phe(gaa)
	3387634	snp	G	T	A	A	614	DNA polymerase III subunit gamma/tau
	3387647	snp	C	A	R	R	615	DNA polymerase III subunit gamma/tau
	3387641	snp	T	G	S	P	617	DNA polymerase III subunit gamma/tau
	3387643	snp	C	A	S	P	617	DNA polymerase III subunit gamma/tau
	3387652	snp	G	C	A	A	620	DNA polymerase III subunit gamma/tau
	3516093	snp	A	T	F	L	1097	Uncharacterized protein YehI
MG1655:	189645	snp	A	C	Q	H	503	UDP-forming cellulose synthase catalytic subunit
dnaX:L635P	337331	snp	G	C	R	S	559	translation elongation factor 4
/D636G	1890031	snp	G	A	G	D	25	manganese efflux pump MntP
	3077921	snp	C	A	I	L	54	Putative hydratase
	1653092	snp	G	C	R	P	35	tRNA dihydrouridine(16) synthase DusC
	1709090	snp	T	G	G	G	94	PTS galactitol transporter subunit IIB
	3387672	snp	T	C	L	P	635	DNA polymerase III subunit gamma/tau
	3387673	snp	G	C	L	P	635	DNA polymerase III subunit gamma/tau
	3387675	snp	A	G	D	G	636	DNA polymerase III subunit gamma/tau
	3387676	snp	T	G	D	G	636	DNA polymerase III subunit gamma/tau

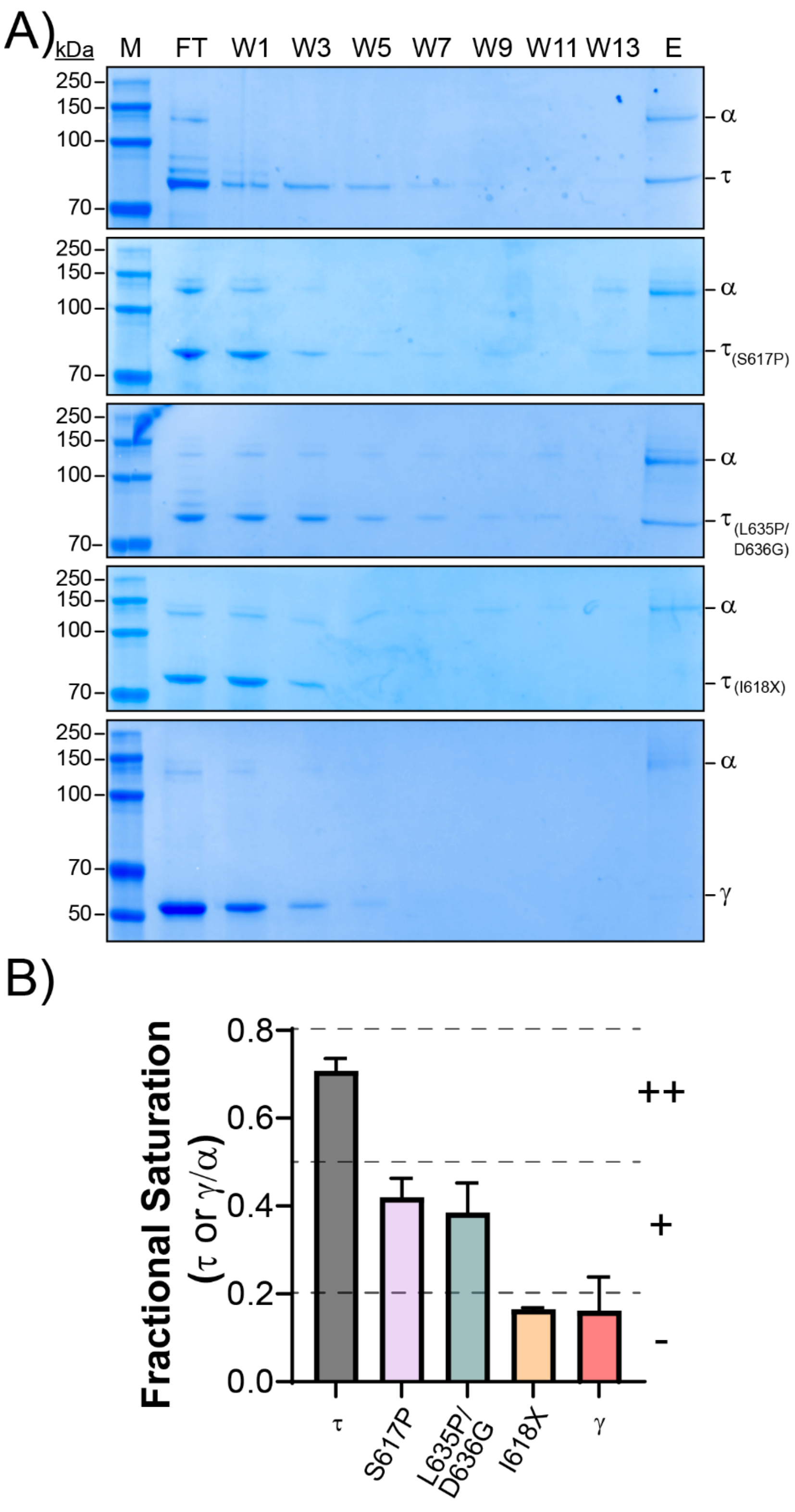

Supporting Figure S1. Pol III α pull-down of τ. A) N-terminally His-tagged α was immobilized onto Ni-NTA resin and τ-CLC (WT, S617P, L635P/D636G, I618X or γ_3-CLC) was added. After the flow through (FT), 13 successive washes were performed to ensure all unbound protein was removed. Finally, His-α and any interacting γ/τ-CLC was eluted (E) with imidazole. Samples were electrophoresed on a 10% Bis-Tris acrylamide gel, stained with SimplyBlue, and visualized with a BioRad Gel Doc EZ imager. B) The fractional saturation ratio of the band intensities for α and τ were used to quantify the interaction strength according to ++, +, and - regions.

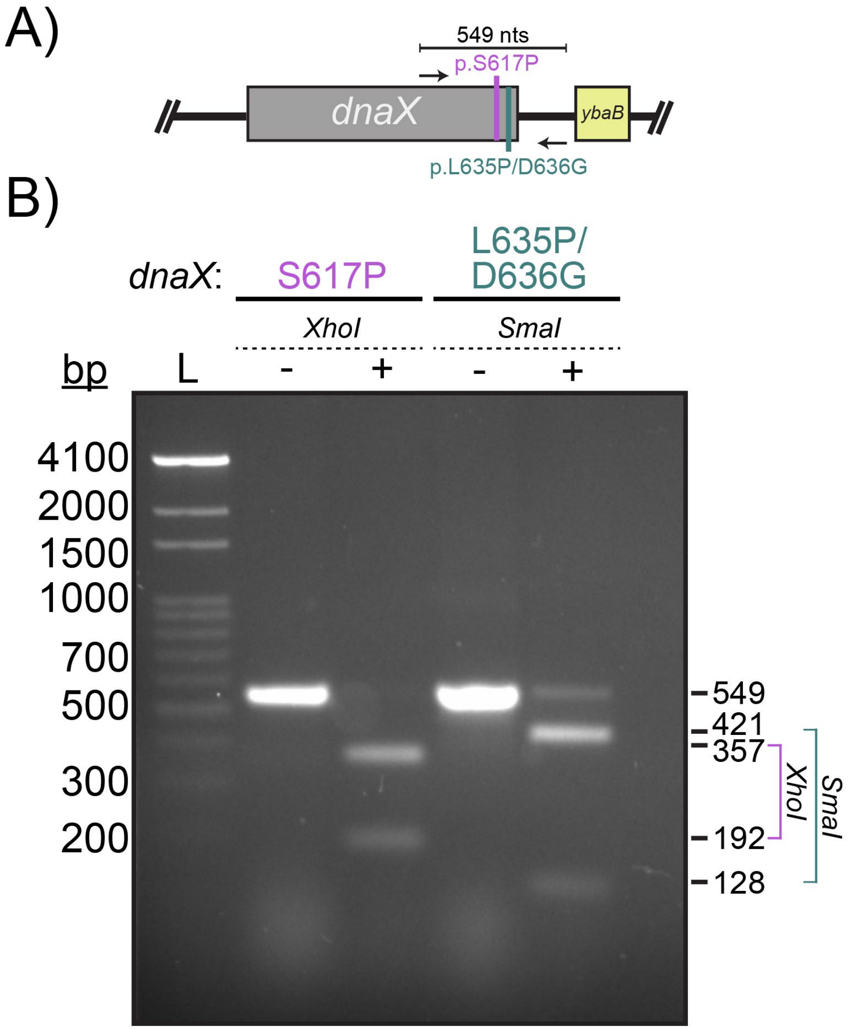

Supporting Figure S2. Restriction digest verification of successful *dnaX* gene editing. A) Schematic of chromosomal region of *dnaX* gene with the adjacent gene, *ybaB*. The locations of the targeted mutation sites are indicated in respective colors (*S617P* - lavender and *L635P/D636G* - teal) and the location of the primers designed to amplify the target region are indicated by arrows. B) A chromosomal region (~549 bp) was amplified by PCR and digested by the respective restriction enzymes (*XhoI* for *dnaX:S617P* and *SmaI* for *dnaX:L635P/D636G*). The band sizes expected from *XhoI* digestion are ~ 360 and ~ 200 bp, and from *SmaI* digestion are ~420 bp and ~ 130 bp.

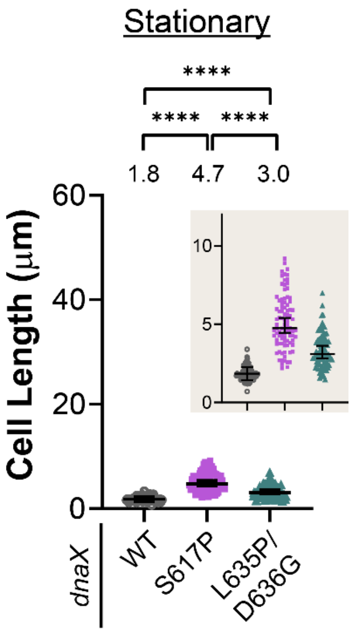

Supporting Figure S3. **Cell filamentation extends into stationary phase.** Cell lengths were measured (n > 80) and median values of cells in stationary phase and plotted. The inset in highlights the range from 0-12 μm. The error bars denote 95% confidence limits, and black bars indicate the statistically significant differences from *P*-values of **** <0.0001.

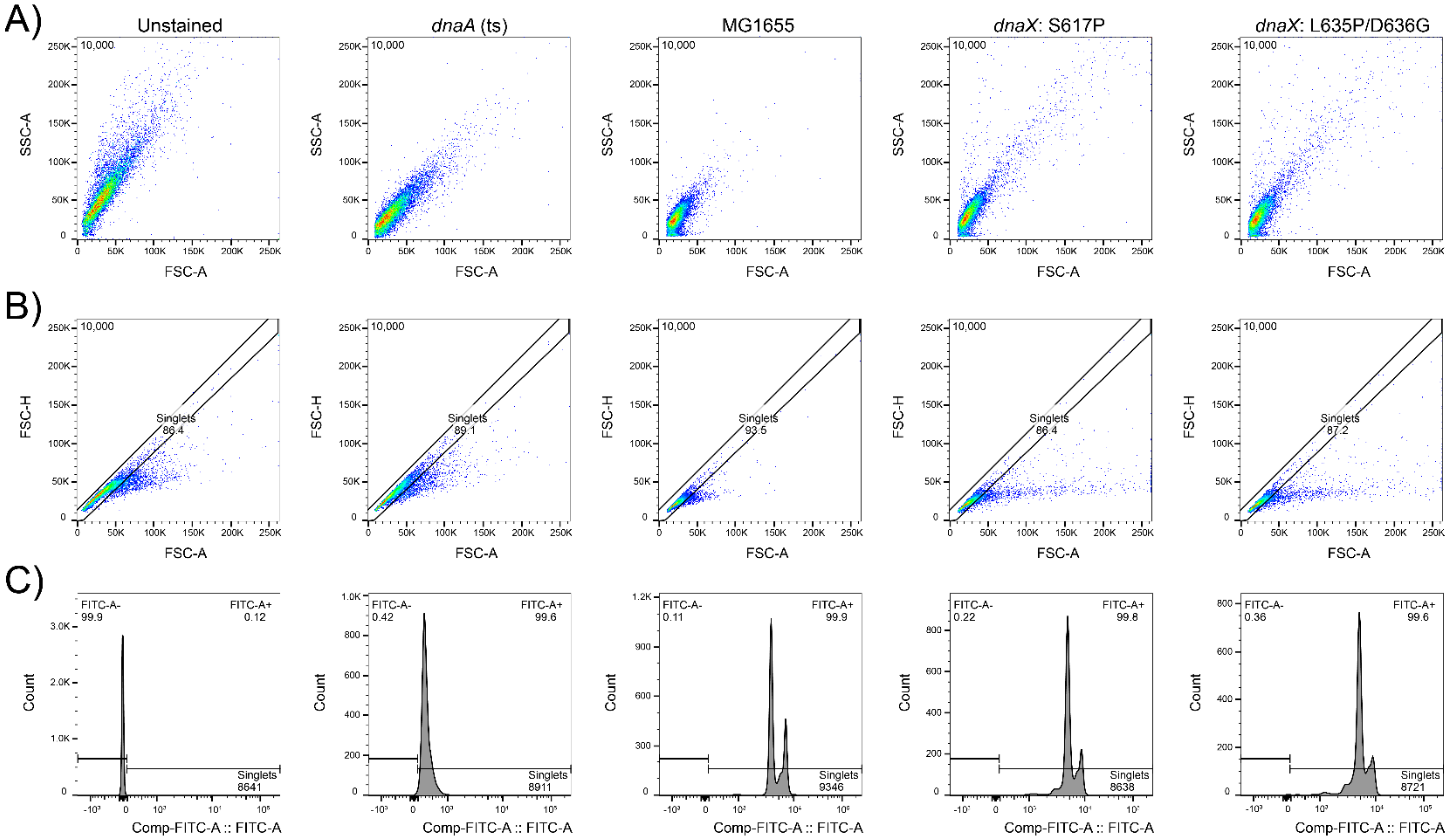

Supporting Figure S4. Peak distribution of chromosomes by FACS. Exponential populations of *dnaX*:*mut* strains were treated with rifampicin and cephalexin in a chromosome and cellular runout assay. A) The top row displays a plot of the FSC (forward scatter) versus SSC (side scatter) (n = 10 000 events). Density of the cell population is represented by color, red being the most dense and dark blue being the least dense. B) The middle row displays scatterplots of FSC-A (area) versus FSC-H (height) and cells are gated for singlets. C) The bottom row shows gated histograms for SytoxGreen stained chromosomes. *dnaA(ts)* strain is used as a control for single chromosome at the non-permissive temperature.

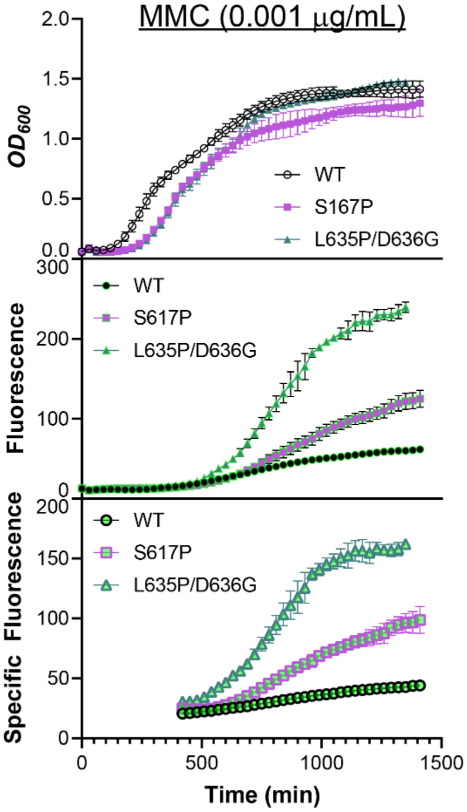

Supporting Figure S5. *dnaX*:*mut* strains induce the SOS response in the presence of low dose MMC. The growth (OD_{600}) and fluorescence (ex 474 nm / em 509 nm) from induction of sgGFP was monitored for *dnaX:mut* strains in Miller LB media using a plate reader at 37 °C with a low dose of MMC (0.001 μg/mL), allowing the calculation of specific fluorescence (bottom panel) using **Eq. 4**.

A)

B)

C)

D)

E)

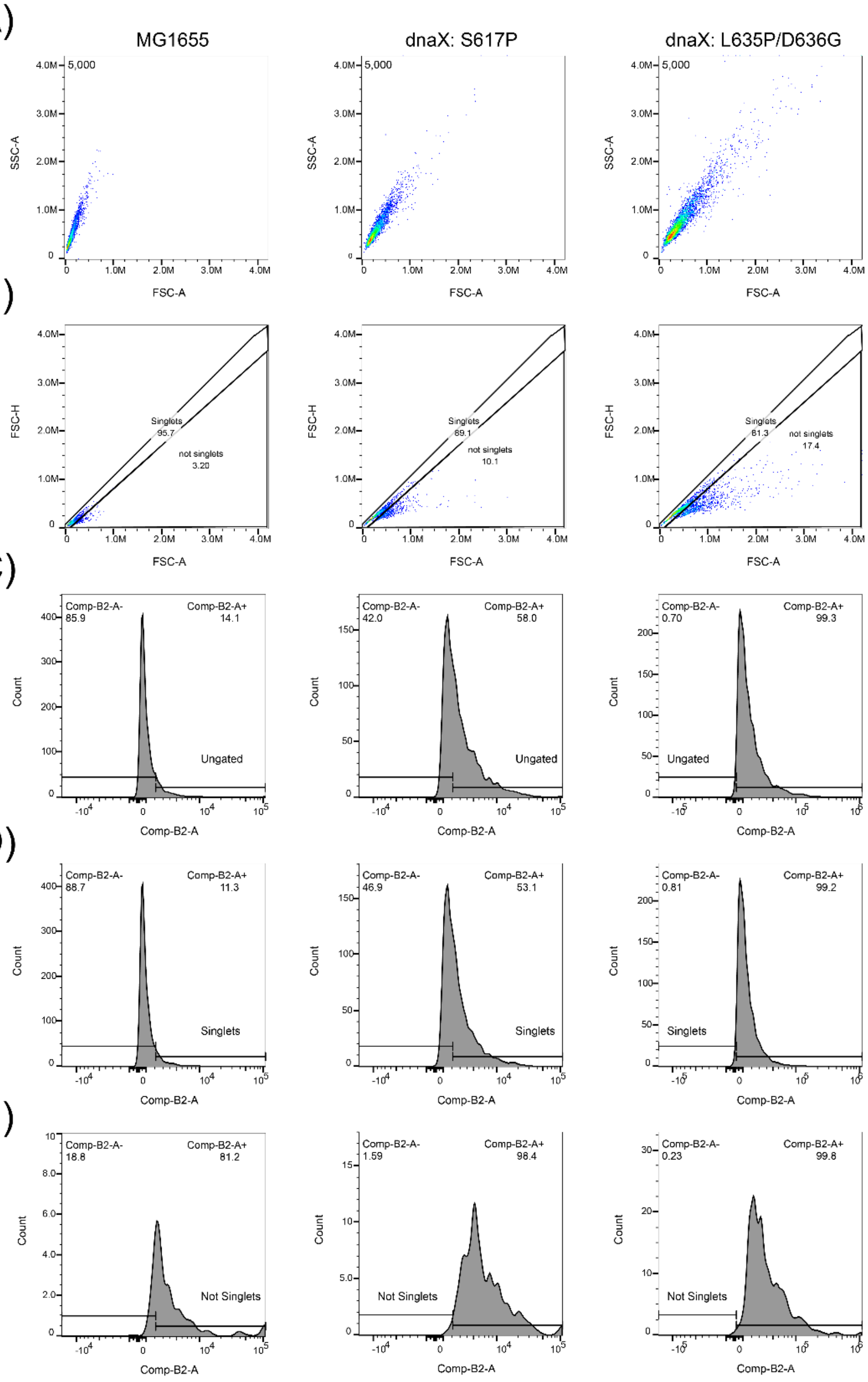

Supporting Figure S6. FACS Analysis of SOS in cell populations by GFP expression. Stationary populations of *dnaX:mut* strains transformed with SuperGlo GFP plasmid were analyzed by FACS. A) The top row displays a plot of the FSC (forward scatter) versus SSC (side scatter) (n = 5 000events). Density of the cell population is represented by color, red being the most dense and dark blue being the least dense. B) Displays scatterplots of FSC-A (area) versus FSC-H (height) and cells are gated for singlets and not singlets (elongated). Gated histograms for SuperGlo GFP for C) all cells, D) singlets, and E) not singlets (elongated).

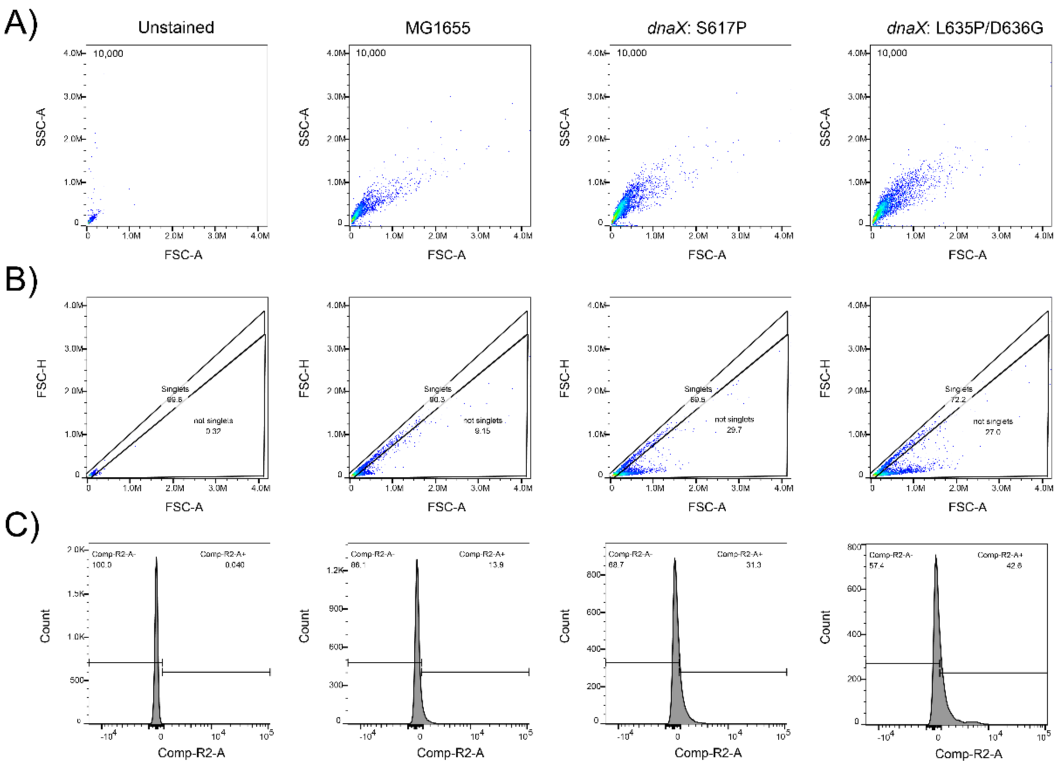

Supporting Figure S7. *dnaX:mut* strains have a larger population of filamented cells and increased ssDNA gaps. Cells in their exponential phase were fixed and treated in a PLUG assay that adds BrdU to single strand gaps. BrdU fluorescence represents ssDNA gaps and was quantified by FACS. A) The top row displays FSC (forward scatter) versus SSC (side scatter) (n=10 000 events). Density of the cell population is represented by color, red being the most dense and dark blue being the least dense. B) The second row shows the gated populations of singlets or not singlets (elongated). C) Histograms show the ungated BrdU positive population.

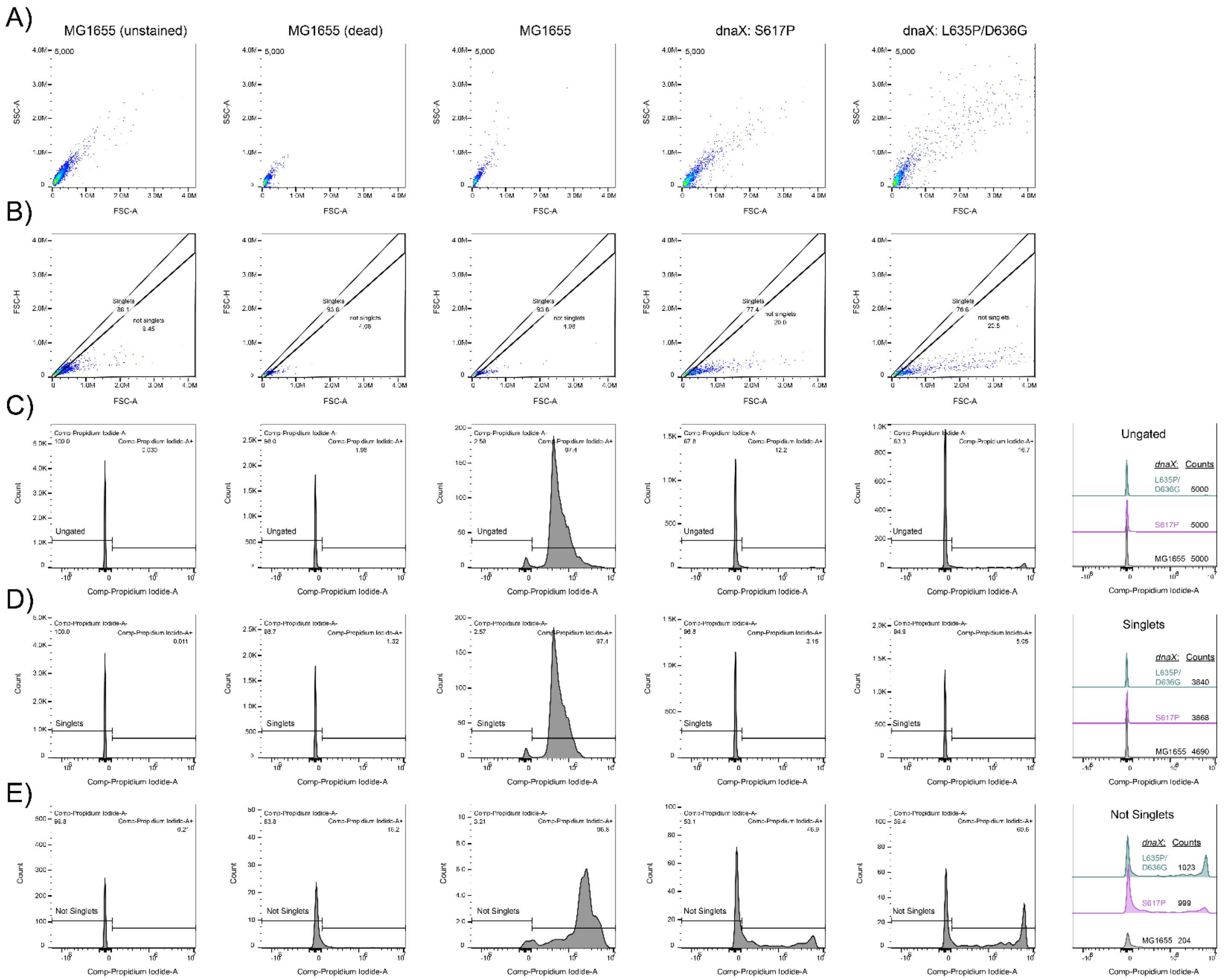

Supporting Figure S8. Live/dead FACS viability assay. Late exponential phase cells of *dnaX*:mut strains were treated with propidium iodide (PI) and analyzed by FACS (n = 5 000 events). Unstained and ethanol treated dead MG1655 cells were used as controls. A) The top row displays scatter plot of FSC (forward scatter) versus SSC (side scatter). Density of the cell population is represented by color, red being the most dense and dark blue being the least dense. B) The second row displays FSC-A (area) versus FSC-H (height) and cells are gated for singlets and not singlets. C) The third row shows ungated histograms using unstained and fixed/stained populations as boundaries for live and dead cells. The D) fourth and E) fifth rows show singlet and not singlet gated histograms of dead cell populations.

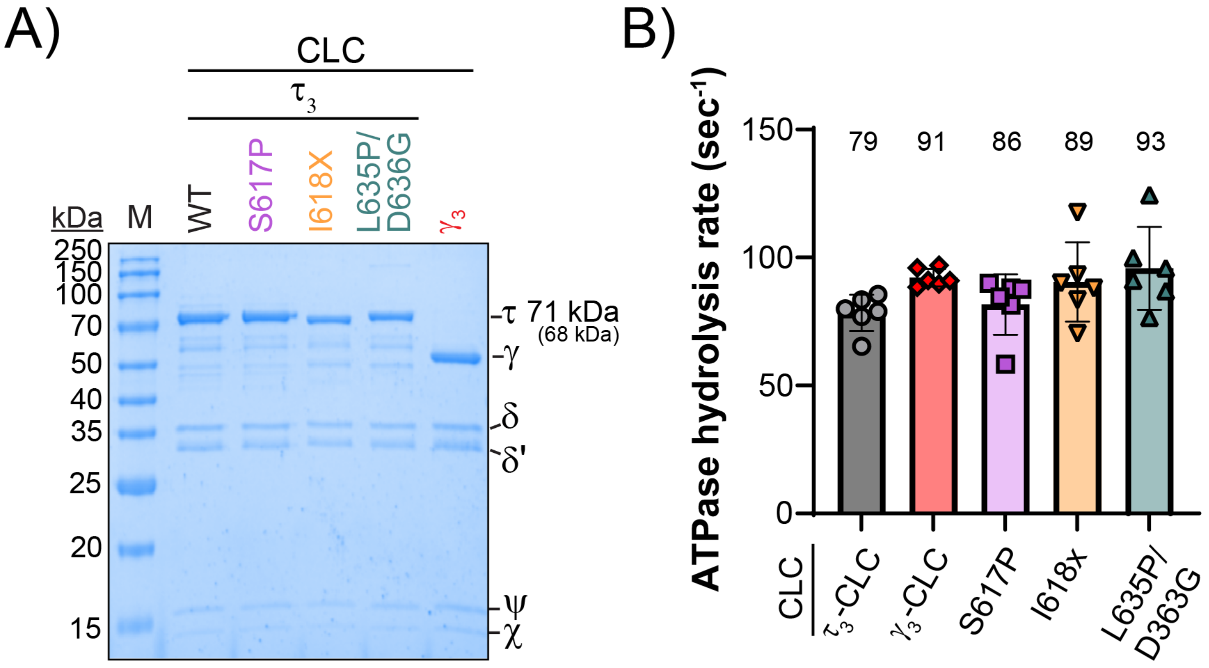

Supporting Figure S9. Purified τ_3-CLC mutant complexes have similar ATP dependent β-clamp loading abilities. A) SDS PAGE of purified CLC ($\tau_3\delta\delta'\psi\chi$) wild-type and mutants. The overexpressed and purified samples were run on a 12% SDS-PAGE gel with the molecular weights indicated. Truncated τ (I618X) has a molecular weight of 68 kDa. B) To verify the activity of the CLC wild-type and mutant proteins, NADH-coupled ATPase assay was performed and the decrease in fluorescence of NADH was measured. The steepest slopes were measured for each of the τ_3-CLCs and plotted with the average ATPase rates indicated. Error bars represent the SE.

References

1. Kim, T., Chitteni-Pattu, S., Cox, B. L., Wood, E. A., Sandler, S. J., and Cox, M. M. (2015) Directed evolution of RecA variants with enhanced capacity for conjugational recombination. *PLoS genetics* **11**, e1005278
2. Chen, S. H., Byrne, R. T., Wood, E. A., and Cox, M. M. (2015) *Escherichia coli radD* (*yejH*) gene: A novel function involved in radiation resistance and double-strand break repair. *Mol. Microbiol.* **95**, 754-768
3. Monachino, E., Ghodke, H., Spinks, R. R., Hoatson, B. S., Jergic, S., Xu, Z. Q. *et al.* (2018) Design of DNA rolling-circle templates with controlled fork topology to study mechanisms of DNA replication. *Anal. Biochem.* **557**, 42-45
4. Naufer, M. N., Murison, D. A., Rouzina, I., Beuning, P. J., and Williams, M. C. (2017) Single-molecule mechanochemical characterization of *E. coli* Pol III core catalytic activity. *Protein Sci.* **26**, 1413-1426
5. Carney, S. M., Gomathinayagam, S., Leuba, S. H., and Trakselis, M. A. (2017) Bacterial DnaB helicase interacts with the excluded strand to regulate unwinding. *J. Biol. Chem.* **292**, 19001-19012
6. Tondnevis, F., Weiss, T. M., Matsui, T., Bloom, L. B., and McKenna, R. (2016) Solution structure of an "open" *E. coli* Pol III clamp loader sliding clamp complex. *J. Struct. Biol.* **194**, 272-281
7. Behrmann, M. S., Perera, H. M., Hoang, J. M., Venkat, T. A., Visser, B. J., Bates, D., and Trakselis, M. A. (2021) Targeted chromosomal *Escherichia coli:dnaB* exterior surface residues regulate DNA helicase behavior to maintain genomic stability and organismal fitness. *PLoS genetics* **17**, e1009886
